# Influence of vitamin D supplementation on muscle strength and exercise capacity in Mongolian schoolchildren: secondary outcomes from a randomised controlled trial

**DOI:** 10.1136/bmjsem-2024-002018

**Published:** 2024-09-26

**Authors:** Davaasambuu Ganmaa, Stephanie Hemmings, David A Jolliffe, Uyanga Buyanjargal, Gantsetseg Garmaa, Unaganshagai Adiya, Tumenulzii Tumurbaatar, Khulan Dorjnamjil, Enkhtsetseg Tserenkhuu, Sumiya Erdenenbaatar, Enkhjargal Tsendjav, Nomin Enkhamgalan, Chuluun-Erdene Achtai, Yagaantsetseg Talhaasuren, Tuya Byambasuren, Erdenetuya Ganbaatar, Erkhembulgan Purevdorj, Adrian R Martineau

**Affiliations:** 1 Harvard Medical School, Boston, Massachusetts, USA; 2 Harvard TH Chan School of Public Health, Boston, Massachusetts, USA; 3 University of Brighton, Brighton, UK; 4 Blizard Institute, Queen Mary University of London, London, UK; 5 Mongolian Health Initiative, Ulaanbaatar, Mongolia; 6 Global Laboratory, Ulaanbaatar, Mongolia; 7 Mongolia Ministry of Health, Ulaanbaatar, Mongolia; 8 Mongolian National University of Medical Sciences, Ulaanbaatar, Mongolia

**Keywords:** Nutrition, Fitness testing, Children's health and exercise

## Abstract

**Objective:**

To determine whether weekly oral vitamin D supplementation influences grip strength, explosive leg power, cardiorespiratory fitness or spirometric lung volumes in Mongolian schoolchildren.

**Methods:**

Multicentre, randomised, placebo-controlled clinical trial conducted in children aged 6–13 years at baseline attending 18 schools in Ulaanbaatar. The intervention was weekly oral doses of 14 000 IU vitamin D_3_ (n=4418) or placebo (n=4433) for 3 years. Outcome measures were grip strength, standing long jump distance and serum 25-hydroxyvitamin D (25(OH)D) concentrations (determined in all participants), peak oxygen uptake (VO_2peak_, determined in a subset of 632 participants using 20 m multistage shuttle run tests) and spirometric outcomes (determined in a subset of 1343 participants).

**Results:**

99.8% of participants had serum 25(OH)D concentrations <75 nmol/L at baseline, and mean end-study 25(OH)D concentrations in children randomised to vitamin D versus placebo were 77.4 vs 26.7 nmol/L (mean difference 50.7 nmol/L, 95% CI 49.7 to 51.4). However, vitamin D supplementation did not influence mean grip strength, standing long jump distance, VO_2peak_, spirometric lung volumes or peak expiratory flow rate, either overall or within subgroups defined by sex, baseline 25(OH)D concentration <25 vs ≥25 nmol/L or calcium intake <500 vs ≥500 mg/day.

**Conclusion:**

A 3-year course of weekly oral supplementation with 14 000 IU vitamin D_3_ elevated serum 25(OH)D concentrations in Mongolian schoolchildren with a high baseline prevalence of vitamin D deficiency. However, this intervention did not influence grip strength, explosive leg power, peak oxygen uptake or spirometric lung volumes, either overall or in subgroup analyses.

**Trial registration number:**

NCT02276755.

**WHAT IS ALREADY KNOWN ON THIS TOPIC**

- Observational studies have reported that vitamin D deficiency associates with reduced muscle strength and peak oxygen uptake in children, but randomised controlled trials (RCT) of vitamin D supplementation to improve grip strength and cardiorespiratory fitness in this age group have yielded conflicting results.

**WHAT THIS STUDY ADDS**

- This phase 3 multicentre RCT of vitamin D supplementation, conducted in Mongolian schoolchildren with a high baseline prevalence of asymptomatic vitamin D deficiency, found that a 3-year course of weekly oral supplementation with 14 000 IU vitamin D_3_ was effective in elevating serum 25-hydroxyvitamin D concentrations. However, this intervention did not influence participants’ grip strength, long jump distance, peak oxygen uptake, spirometric lung volumes or peak expiratory flow rate, either overall or in subgroup analyses.

**HOW THIS STUDY MIGHT AFFECT RESEARCH, PRACTICE OR POLICY**

- Taken together with results from another phase 3 randomised controlled trial of vitamin D supplementation conducted in South African children, our findings do not suggest a role for weekly oral vitamin D supplementation to enhance muscle strength, peak oxygen uptake or respiratory function in schoolchildren in whom rickets has been excluded.

## Introduction

 Muscular strength and exercise tolerance in childhood are positive correlates of physical and mental health that are associate with reduced cardiometabolic risk later in life.^1^,^4^ Vitamin D plays a key role in supporting normal development and function of skeletal muscle, the lung and the heart.^5^,^7^ Vitamin D deficiency is common among children living in higher-income and lower-income countries alike^8^,^11^ and has been reported to be associated with lower muscle strength, poorer cardiorespiratory fitness and lower spirometric lung volumes in children and adolescents participating in observational studies.^12^,^14^ Numerous randomised controlled trials (RCTs) of vitamin D supplementation to improve muscle strength and power have been conducted in adults, with meta-analysis revealing small positive impacts.^15^ Fewer RCTs have been conducted in children, and these have yielded inconsistent results. One trial in male soccer players in Tunisia aged 8–15 years has reported improvements in jump, sprint and shuttle run outcomes^16^ while another conducted in girls aged 12–14 year-olds in the UK reported a statistically significant improvement in efficiency of movement, with trends towards improvements in jumping velocity and grip strength.^17^ Other RCTs conducted in children and adolescents living in Denmark,^18 19^ the USA,^20^ Israel^21^ and Lebanon^22^ have reported null overall effects for outcomes including grip strength, leg press strength and swimming performance. No such trials have yet been conducted in Asia; moreover, there is a lack of large, multicentre trials examining the impact of prolonged (greater than 1 year) vitamin D supplementation on muscle strength, cardiorespiratory fitness and spirometric outcomes in children with a high baseline prevalence of vitamin D deficiency, regardless of setting.

Given these limitations of the existing evidence base, we took the opportunity to generate new data in this area during the conduct of a phase 3 RCT of vitamin D supplementation in 8851 schoolchildren aged 6–13 years living in Mongolia who were given weekly oral vitamin D supplementation over 3 years.^23^,^25^ The primary aim of the trial was to test whether this intervention reduced the risk of incident tuberculosis infection; null results for this outcome have been reported elsewhere.^23^ This paper reports findings for secondary outcomes that were investigated to test the hypotheses that vitamin D supplementation would improve grip strength and standing long jump distance, measured at annual intervals in all participants; peak oxygen uptake (VO_2peak_), estimated using 20 m shuttle run tests performed at annual intervals in a subset of 632 participants; and spirometric lung volumes, measured at 3-year follow-up in a subset of 1343 participants.

## Methods

### Study design, setting, participants, randomisation and intervention

We conducted a parallel two-arm individually randomised placebo-controlled trial in 18 public schools in Ulaanbaatar, Mongolia, as previously described.^23^,^25^ The study protocol is available in online supplemental file 1. The primary outcome of the trial was the acquisition of latent tuberculosis infection; the current manuscript reports the effects of the intervention on prespecified secondary outcomes relating to grip strength, explosive leg power, peak oxygen uptake and spirometric outcomes. Principal inclusion criteria were age 6–13 years at screening and attendance at a participating school; principal exclusion criteria were a positive QuantiFERON-TB Gold in-tube assay (QFT) result, presence of conditions associated with vitamin D hypersensitivity (primary hyperparathyroidism or sarcoidosis) or immunocompromise (taking immunosuppressant medication or cytotoxic therapy), use of vitamin D supplements, signs of rickets (all participants were screened for rickets via physical examination by a paediatrician), or intention to move from Ulaanbaatar within 4 years of enrolment. Trial staff who determined whether subjects were eligible for inclusion in the trial were unaware which group the next subject would be allocated to, that is, allocation was concealed. Eligible participants were individually randomised to receive a weekly capsule containing 14 000 IU (350 µg) vitamin D_3_ or a weekly capsule of placebo (olive oil) for 3 years, with a one-to-one allocation ratio and stratification by school of attendance. Capsules containing vitamin D_3_ had identical appearance and taste to placebo capsules. The dose was chosen on the basis of results of another RCT showing that a weekly oral administration of 14 000 IU vitamin D_3_ to schoolchildren aged 10–17 years for 1 year resulted in a 52.5 nmol/L increase in mean 25(OH)D concentration without inducing adverse events.^26^

The randomisation list was computer generated by a statistician (Dr Polyna Khudyakov, Sage Therapeutics, Cambridge, Massachusett, USA) prior to the start of recruitment. This list was held by the data monitoring committee during the conduct of the trial: concealment of allocation was achieved by ensuring that no trial staff (including those who assessed eligibility) had access to the trial randomisation list. All participants and their parents/guardians, and all trial personnel, including principal investigators and all those who had contact with study participants, including those who assessed outcomes, were blinded to participant allocation during the conduct of the trial. The trial is registered with ClinicalTrials.gov (NCT02276755).

### Baseline procedures

At baseline, participants’ parents were asked to complete questionnaires detailing their socioeconomic circumstances, lifestyle and dietary factors influencing vitamin D status, and intake of foods previously shown to be major contributors to dietary calcium intake in urban Mongolia.^27^ For all participants, height was then measured using a portable stadiometer (SECA, Hamburg, Germany), weight was measured using a Digital Floor Scale (SECA), grip strength was measured as described elsewhere^28^ using a portable dynamometer (Takei Digital Grip Strength Dynamometer, Model T.K.K.5401, with the best of two readings for the dominant hand recorded, except where injury precluded measurement, where strength of the other hand was measured), and standing long jump distance was measured as described elsewhere^29^ using a DiCUNO measuring tape, with the best of two readings recorded. Validity and reliability of the Takei Digital Grip Strength Dynamometer for measurement of grip strength, and of the standing long jump for measurement of lower body muscular power have both been demonstrated in schoolchildren studied in other settings.^30 31^ In a subset of 620 children who additionally participated in the exercise substudy, a 20 m multistage shuttle run test was administered as described below to determine VO2peak. A blood sample was drawn from all participants at baseline for separation and storage of serum for determination of baseline 25(OH)D concentrations as described below.

### Follow-up procedures and outcomes

During school terms, study participants had weekly face-to-face visits at which study capsules were administered and adverse events were recorded. During school holidays, children were either given a single bolus dose of up to 36 000 IU (shorter holidays), study staff travelled to participants’ homes to administer medication, or parents were supplied with sufficient trial medication to cover the holiday period, along with instructions on its storage and administration. Using the same methods as at baseline, all participants were reassessed at 12-month, 24-month and 36-month follow-ups for grip strength and long jump distance. Exercise substudy participants additionally completed 20 m multistage shuttle run tests at 12-month, 24-month and 36-month follow-up, as described below. Spirometry substudy participants additionally underwent spirometry testing at 36-month follow-up using a portable spirometer (spirolab III, Medical International Research, Rome, Italy) and performed according to ERS/ATS standards^32^ to assess % predicted forced expiratory volume in 1 s (FEV1), % predicted forced vital capacity (FVC), % predicted FEV1/FVC, % predicted peak expiratory flow rate (PEFR) and % predicted forced expiratory flow over the middle one-half of the FVC (FEF25–75).

### Measurement of vitamin D status

25(OH)D concentrations were determined in serum samples from baseline and 3-year follow-up using an enzyme-linked fluorescent assay (VIDAS 25OH Vitamin D total, bioMérieux, Marcy-l’Étoile, France). Non-zero 25(OH)D values were standardised using a published method,^33^ using a set of 40 serum samples provided by DEQAS (the Vitamin D External Quality Assessment Scheme, http://www.deqas.org/). The total coefficient of variation (CV) was 7.9%, the mean bias was 7.7% and the limit of quantitation (LOQ) was 20.2 nmol/L. Values below the LOQ were classified as <20.2 nmol/L.

### Exercise test

A 20 m multistage shuttle run test was conducted using freely available recorded instruction (Shuttle run bleep test, www.bleeptests.com). Two lines were marked 20 m apart and an audible ‘beep’ signalled to participants the speed required to run between them. Participants’ number of completed laps was used to derive their estimated VO_2peak_ using a published formula.^34^

### Sample size and statistical methods

All analyses were conducted by using STATA V.IC 15.1 (StataCorp). The sample size calculation for the main trial was based on the power needed to detect a clinically significant effect of the intervention on the primary endpoint (incident latent tuberculosis infection) as described previously.^20^ For the exercise substudy, assuming an SD for VO_2peak_ of 6.5 mL/kg/min at 3-year follow-up,^34^ we calculated that a total of 334 participants (167 per arm) would need to be recruited and followed up to have 80% power to detect a clinically significant difference of 2 mL/kg/min between arms with 5% alpha. Allowing for a 20% loss to follow-up, we originally estimated that a total of 420 participants (210 per arm) would need to be recruited for the exercise substudy. Subsequently, we became concerned that rates of loss to follow-up might be higher than originally anticipated, and consequently, the target sample size for this substudy was increased to 614. Ultimately, the exercise substudy over-recruited slightly (n=632). Spirometry was performed for a subset of children who participated in a bone health substudy, powered to detect an effect of the intervention on the radial speed of sound Z-scores as described elsewhere.^25^

Estimated calcium intakes were calculated on the basis of parental responses to a food frequency questionnaire, as described elsewhere.^24^ Anthropometric measurements and data on participants’ age and sex were used to compute Z-scores for height-for-age and BMI-for-age, using the Canadian Pediatric Endocrine Group who2007 Shiny App (https://cpeg-gcep.shinyapps.io/who2007_cpeg/) based on WHO 2007 growth reference data for 5–19 years.^35^ Serum 25(OH)D values were adjusted for seasonal variation prior to analysis using a sinusoidal model.^36^ Outcomes measured over multiple years’ follow-up were analysed overall and in each subgroup using mixed models for repeated measures with fixed effects for treatment and time and treatment-by-time interaction, adjusted for school of attendance and random effects for individuals. Adjusted treatment mean differences at different time points are presented with 95% CIs, and significance tests were conducted for the treatment effect at each time point and the overall treatment-by-time interaction. Where overall p values were less than 0.05, we applied a Benjamini Hochberg procedure with a 10% false discovery rate^37^ to the relevant family of p values to adjust for multiple comparisons. Outcomes measured at end-study only were analysed using general linear models, adjusted for school of attendance. Prespecified subgroup analyses were conducted according to participants’ sex (males vs females), baseline deseasonalised 25(OH)D concentration (<25 nmol/L vs ≥25 nmol/L) and estimated calcium intake (<500 mg/day vs ≥500 mg/day). The primary p values for outcome modelling were the overall p values, that is, those associated with the interaction between follow-up time point and treatment allocation.

### Patient and public involvement

We consulted children and their parents/guardians on questionnaire design and acceptability of clinical measurements prior to implementation of the trial. Study findings were disseminated via community engagement events.

### Equity, diversity and inclusion statement

This trial recruited a group of participants who are under-represented in research (schoolchildren in Mongolia). Participation was open to children of any gender, race/ethnicity/culture and socioeconomic level. The author team is gender-balanced and nationality-balanced and includes junior researchers and perspectives from multiple disciplines. Subgroup analyses were conducted to detect gender differences in response to vitamin D supplementation.

## Results

### Participants

**Figure 1:**
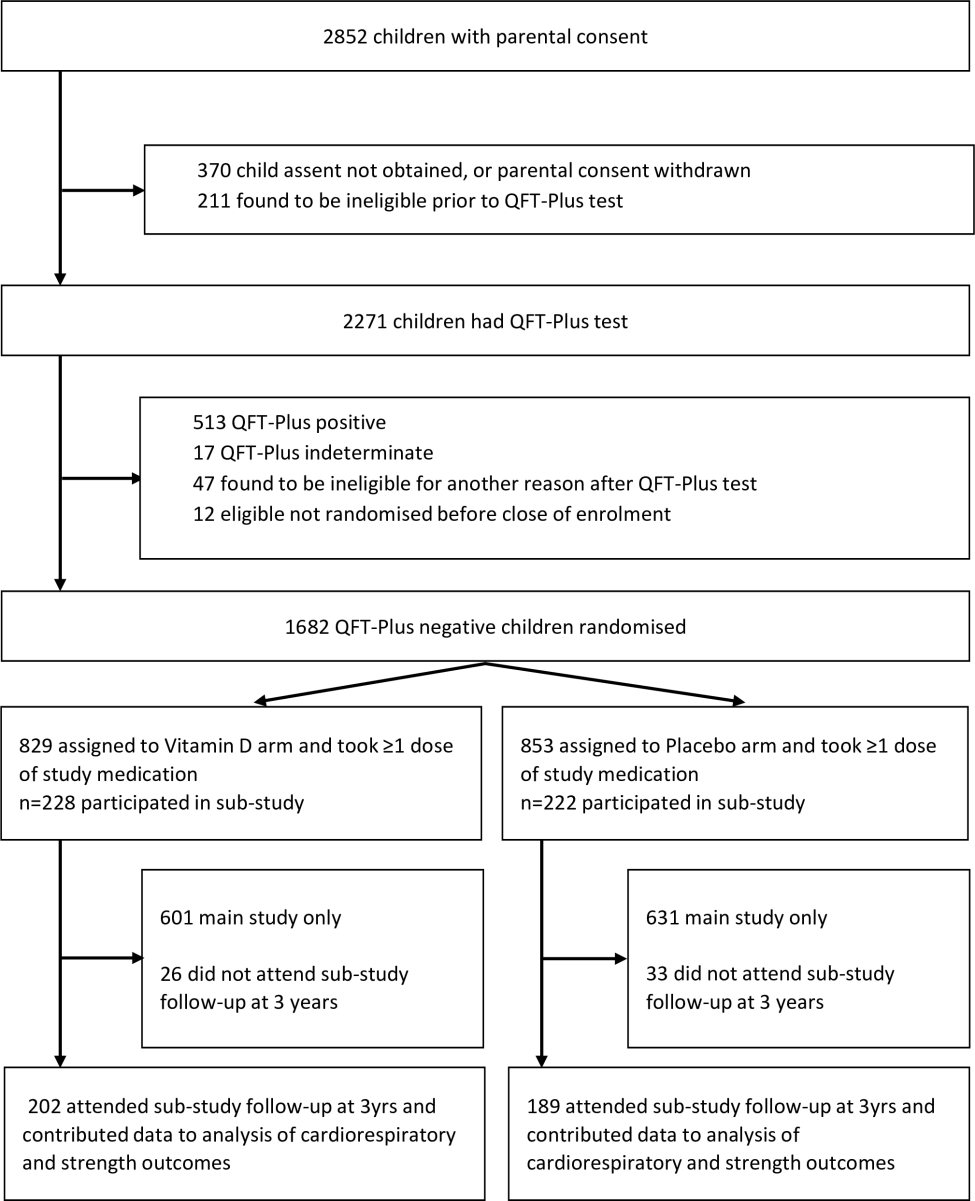
Trial profile. QFT, QuantiFERON-TB Gold in-tube assay.

11 475 children were invited to participate in the study from September 2015 to March 2017 inclusive, of whom 9814 underwent QFT testing: 8851 QFT-negative children were randomly assigned to receive vitamin D or placebo (4418 vs 4433, respectively) as previously described.^23^ Of these, 632 children (302 vs 330 randomised to vitamin D vs placebo, respectively) also participated in the exercise substudy, and 1343 children (666 vs 677 randomised to vitamin D vs placebo, respectively) underwent spirometry at 3-year follow-up (figure 1). Table 1 shows participants’ baseline characteristics: overall, mean age was 9.4 years and 49.3% were female. Mean deseasonalised baseline serum 25(OH)D concentration was 29.7 nmol/L (SD 10.5). Baseline characteristics were well balanced between participants randomised to vitamin D versus placebo, both for those in the main trial and for participants contributing data to the exercise and spirometry substudies. Mean serum 25(OH)D concentration at 3-year follow-up was higher in the vitamin D group versus the placebo group (77.4 vs 26.7 nmol/L, respectively; mean difference 50.7 nmol/L, 95% CI 49.7 to 51.4). 8549 participants contributed data to the analysis of grip strength; 8539 to the analysis of long jump distance; 611 to the analysis of cardiorespiratory fitness and 1343 to the analysis of spirometric outcomes (figure 1).

**Table 1:** Participants’ baseline characteristics by allocation and overall: main trial, exercise substudy and spirometry substudy

	Main trial			Exercise substudy			Spirometry substudy		
	Placebo	Vitamin D	Total	Placebo	Vitamin D	Total	Placebo	Vitamin D	Total
	N=4433	N=4418	N=8851	N=330	N=302	N=632	N=677	N=666	N=1343
Mean age, years (SD) (range)	9.4 (1.6)(5.9–13.7)	9.4 (1.6)(5.8–14.2)	9.4 (1.6)(5.8–14.2)	9.5 (1.5)(6.5–12.8)	9.5 (1.5)(5.8–13.9)	9.5 (1.5)(5.8–13.9)	9.5 (1.5)(6.5–13.0)	9.5 (1.6)(6.4–13.3)	9.5 (1.6)(6.4–13.3)
Sex, n (%)									
Female	2224 (50.2)	2142 (48.5)	4366 (49.3)	166 (50.3)	141 (46.7)	307 (48.6)	325 (48.0)	333 (50.0)	658 (49.0)
Male	2209 (49.8)	2276 (51.5)	4485 (50.7)	164 (49.7)	161 (53.3)	325 (51.4)	352 (52.0)	333 (50.0)	685 (51.0)
Ethnic origin									
Khalkh, n (%)	4103 (92.6)	4062 (91.9)	8165 (92.2)	300 (90.9)	275 (91.1)	575 (91.0)	628 (92.8)	607 (91.1)	1235 (92.0)
Other, n (%)	330 (7.4)	356 (8.1)	686 (7.8)	30 (9.1)	27 (8.9)	57 (9.0)	49 (7.2)	59 (8.9)	108 (8.0)
Parental education*									
Secondary school or lower, n (%)	2457 (55.4)	2401 (54.3)	4858 (54.9)	201 (60.9)	171 (56.6)	372 (58.9)	417 (61.6)	398 (59.8)	815 (60.7)
University/polytechnic, n (%)	1976 (44.6)	2017 (45.7)	3993 (45.1)	129 (39.1)	131 (43.4)	260 (41.1)	260 (38.4)	268 (40.2)	528 (39.3)
Type of residence									
House with central heating, n (%)	1083 (24.4)	1110 (25.1)	2193 (24.8)	62 (18.8)	54 (17.9)	116 (18.4)	100 (14.8)	99 (14.9)	199 (14.8)
Ger (Yurt), n (%)	1628 (36.7)	1643 (37.2)	3271 (37.0)	112 (33.9)	106 (35.1)	218 (34.5)	277 (40.9)	290 (43.5)	567 (42.2)
House without central heating, n (%)	1722 (38.8)	1665 (37.7)	3387 (38.3)	156 (47.3)	142 (47.0)	298 (47.2)	300 (44.3)	277 (41.6)	577 (43.0)
Home ownership, n (%)									
No	963 (21.7)	925 (20.9)	1888 (21.3)	77 (23.3)	55 (18.2)	132 (20.9)	164 (24.2)	157 (23.6)	321 (23.9)
Yes	3470 (78.3)	3493 (79.1)	6963 (78.7)	253 (76.7)	247 (81.8)	500 (79.1)	513 (75.8)	509 (76.4)	1022 (76.1)
Mean monthly household income, US dollars (SD)†	845.8 (603.7)	850.6 (553.8)	848.2 (579.3)	769.5 (411.6)	844.1 (515.3)	805.1 (465.2)	751.5 (441.2)	778.0 (427.8)	764.6 (434.7)
Calcium intake†									
<500 mg/day, n (%)	2628 (64.6)	2599 (63.7)	5227 (64.2)	190 (63.8)	179 (63.3)	369 (63.5)	423 (63.4)	435 (66.1)	858 (64.8)
≥500 mg/day, n (%)	1437 (35.4)	1481 (36.3)	2918 (35.8)	108 (36.2)	104 (36.7)	212 (36.5)	244 (36.6)	223 (33.9)	467 (35.2)
Mean BMI-for-age z-score (SD)†	0.2 (1.1)	0.2 (1.0)	0.2 (1.1)	0.2 (1.1)	0.1 (1.1)	0.2 (1.1)	0.1 (1.0)	0.1 (1.1)	0.1 (1.0)
Mean height-for-age z-score (SD)†	−0.3 (1.0)	−0.3 (1.0)	−0.3 (1.0)	−0.3 (1.0)	−0.3 (1.1)	−0.3 (1.0)	−0.4 (0.9)	−0.3 (1.0)	−0.4 (1.0)
Mean serum 25(OH)D, nmol/L (SD)^2 3^	29.7 (10.5)	29.5 (10.5)	29.7 (10.5)	28.6 (11.5)	27.5 (10.5)	28.1 (11.0)	28.5 (10.5)	28.1 (10.7)	28.3 (10.6)
Serum 25(OH)D category†‡									
<25 nmol/L, n (%)	1424 (32.1)	1395 (31.6)	2819 (31.8)	126 (38.2)	116 (38.4)	242 (38.3)	253 (37.4)	232 (34.8)	485 (36.1)
25–49.9 nmol/L, n (%)	2809 (63.4)	2827 (64.0)	5636 (63.7)	189 (57.3)	174 (57.6)	363 (57.4)	399 (58.9)	408 (61.3)	807 (60.1)
50–74.9 nmol/L, n (%)	193 (4.4)	187 (4.2)	380 (4.3)	14 (4.2)	11 (3.6)	25 (4.0)	25 (3.7)	26 (3.9)	51 (3.8)
≥75 nmol/L, n (%)	7 (0.2)	9 (0.2)	16 (0.2)	1 (0.3)	1 (0.3)	2 (0.3)	–	–	–
Mean maximal grip strength, kg (SD)†	12.0 (4.0)	12.0 (4.0)	12.0 (4.0)	12.2 (3.9)	12.4 (3.8)	12.3 (3.9)	12.0 (3.9)	12.3 (4.1)	12.1 (4.0)
Mean standing long jump distance, m (SD)†	107.9 (21.8)	108.2 (22.6)	108.0 (22.2)	106.7 (21.6)	108.8 (21.2)	107.7 (21.5)	107.4 (21.7)	107.9 (23.0)	107.6 (22.4)
Mean VO_2peak_, mL/kg/min (SD)†	–	–	–	43.8 (3.1)	44.2 (3.3)	44.0 (3.2)	–	–	–

* Highest educational level attained by either parent;.

† Missing values: monthly household income missing for 2 participants in the main trial (1 randomised to placebo, 1 to vitamin D); calcium intake missing for 706 participants in the main trial (368 randomised to placebo, 338 to vitamin D), 51 participants in the exercise substudy (32 randomised to placebo, 19 to vitamin D) and 18 participants in the spirometry substudy (18 randomised to placebo, 10 to vitamin D); BMI-for-age z-score missing for one1 participant in the main trial (randomised to placebo); height-for-age z-score missing for one1 participant in the main trial (randomised to placebo); 25(OH)D missing for 5 participants in the main trial (4 randomised to placebo, and 1 to vitamin D); grip strength missing for 6 participants in the main trial (2 randomised to placebo, 4 to vitamin D) and 1 participant in the spirometry substudy (randomised to placebo); long jump distance missing for 38 participants in the main trial (23 randomised to placebo, 15 to vitamin D), 2 participants in the exercise substudy (both randomised to placebo) and 3 participants in the spirometry substudy (2 randomised to placebo, and 1 to vitamin D); VO2 peak missing for 17 participants in the exercise substudy (11 randomised to placebo, and 6 to vitamin D).

‡ Baseline 25(OH)D values deseasonalised.

BMIbody mass index25(OH)D25-hydroxyvitamin DSOSspeed of soundVO_2peak_peak oxygen consumption

### Outcomes

Allocation to vitamin D versus placebo did not influence mean grip strength, either overall or in subgroups defined by male versus female sex, baseline 25(OH)D concentration <25 vs ≥25 nmol/L or estimated calcium intake <500 vs ≥500 mg/day (table 2). Similarly, no effect of the intervention was seen on long jump distance, either overall or by subgroup, after correction for multiple comparison testing (table 3). Among exercise substudy participants, allocation to vitamin D versus placebo did not influence mean VO_2peak_, either overall or within subgroups defined by male versus female sex, baseline 25(OH)D concentration<25 vs ≥25 nmol/L or estimated calcium intake <500 vs ≥500 mg/day (table 4). Among participants who underwent spirometry at 3-year follow-up, allocation to vitamin D versus placebo did not influence % predicted FEV1, FVC, FEV1/FVC, PEFR or FEF25–75 after correction for multiple comparison testing, either overall or within subgroups defined by male versus female sex, baseline 25(OH)D concentration <25 vs ≥25 nmol/L or estimated calcium intake <500 vs ≥500 mg/day (table 5).

**Table 2:** Mean grip strength in main trial participants at 1, 2 and 3 years follow-up by allocation: overall and by subgroup

	Vitamin D arm: mean strength, kg (SD) (n)	Placebo arm: mean strength, kg (SD) (n)	Adjusted mean difference (95% CI)*	P for time point	Overall p value	P for interaction
Overall						
1 year	15.0 (4.9) (4219)	14.9 (4.8) (4229)	0.03 (−0.19, 0.25)	0.79	0.999	--
2 years	17.1 (5.5) (3802)	17.3 (5.5) (3829)	−0.09 (−0.31, 0.13)	0.41		
3 years	20.0 (6.4) (4074)	20.0 (6.4) (4054)	0.01 (−0.21, 0.23)	0.94		
By sex						
Male						
1 year	15.6 (5.0) (2184)	15.5 (4.8) (2103)	0.10 (−0.22, 0.42)	0.53	0.90	0.55
2 years	17.9 (5.8) (1952)	18.0 (5.8) (1902)	−0.07 (−0.40, 0.26)	0.67		
3 years	21.4 (7.2) (2100)	21.4 (7.1) (2010)	−0.00 (−0.32, 0.32)	0.99		
Female						
1 year	14.2 (4.7) (2035)	14.3 (4.7) (2126)	−0.10 (−0.38, 0.18)	0.47	0.48	
2 years	16.2 (5.1) (1850)	16.5 (5.1) (1927)	−0.19 (−0.47, 0.09)	0.19		
3 years	18.6 (5.1) (1974)	18.7 (5.2) (2044)	−0.10 (−0.38, 0.18)	0.5		
By baseline 25(OH)D concentration†						
<25 nmol/L						
1 year	15.5 (5.2) (1342)	15.5 (5.3) (1352)	0.05 (−0.33, 0.44)	0.78	0.73	0.75
2 years	17.7 (5.9) (1214)	17.9 (6.0) (1226)	−0.05 (−0.44, 0.34)	0.81		
3 years	20.4 (6.7) (1291)	20.5 (6.7) (1309)	0.14 (−0.24, 0.53)	0.47		
≥25 nmol/L						
1 year	14.7 (4.7) (2876)	14.6 (4.5) (2873)	0.08 (−0.17, 0.33)	0.53	0.77	
2 years	16.8 (5.3) (2587)	16.9 (5.3) (2600)	−0.05 (−0.31, 0.21)	0.71		
3 years	19.8 (6.3) (2782)	19.8 (6.2) (2742)	0.02 (−0.24, 0.27)	0.91		
By calcium intake						
<500 mg/day						
1 year	15.0 (4.8) (1463)	14.9 (4.8) (1422)	0.12 (−0.16, 0.40)	0.41	0.51	0.20
2 years	17.2 (5.6) (1355)	17.2 (5.6) (1305)	−0.00 (−0.29, 0.29)	0.998		
3 years	20.2 (6.4) (1444)	20.0 (6.5) (1410)	0.18 (−0.10, 0.46)	0.21		
≥500 mg/day						
1 year	14.8 (5.0) (2572)	15.0 (4.7) (2596)	0.12 (−0.16, 0.40)	0.41	0.38	
2 years	16.8 (5.5) (2336)	17.3 (5.5) (2400)	−0.00 (−0.29, 0.29)	0.998		
3 years	19.7 (6.5) (2549)	20.1 (6.2) (2561)	0.18 (−0.10, 0.46)	0.21		

* Adjusted for baseline value and school of attendance.

† Deseasonalised values.

Nnumber25(OH)D25-hydroxyvitamin D

**Table 3:** Mean long jump distance in main trial participants at 1, 2 and 3 years follow-up by allocation: overall and by subgroup

	Vitamin D arm: mean distance, m (SD) (n)	Placebo arm: mean distance, m (SD) (n)	Adjusted mean difference (95% CI)*	P for time point	Overall p value	P for interaction
Overall						
1 year	117.6 (22.4) (4206)	117.1 (21.9) (4201)	0.47 (−0.50, 1.45)	0.34	0.33	--
2 years	124.2 (23.5) (3790)	124.0 (22.8) (3809)	0.42 (−0.57, 1.41)	0.41		
3 years	132.0 (26.4) (4012)	131.3 (25.4) (3988)	0.76 (−0.22, 1.74)	0.13		
By sex						
Male						
1 year	125.9 (22.2) (2177)	124.9 (21.6) (2094)	0.71 (−0.66, 2.08)	0.31	0.17	0.04†
2 years	134.2 (23.7) (1944)	133.1 (23.0) (1895)	1.10 (−0.30, 2.50)	0.13		
3 years	144.4 (26.9) (2065)	142.9 (25.8) (1984)	1.32 (−0.06, 2.70)	0.06		
Female						
1 year	108.8 (18.9) (2029)	109.4 (19.2) (2107)	−0.40 (−1.53, 0.72)	0.48	0.18	
2 years	113.6 (18.0) (1846)	115.0 (18.6) (1914)	−1.01 (−2.17, 0.14)	0.09		
3 years	118.8 (18.3) (1947)	119.9 (18.9) (2004)	−0.73 (−1.87, 0.41)	0.21		
By baseline 25(OH)D concentration‡						
<25 nmol/L						
1 year	119.2 (22.6) (1339)	119.3 (22.1) (1343)	0.32 (−1.42, 2.05)	0.72	0.78	0.54
2 years	125.6 (24.1) (1212)	126.1 (23.9) (1219)	0.51 (−1.26, 2.28)	0.58		
3 years	133.3 (26.8) (1268)	133.3 (25.4) (1288)	0.80 (−0.96, 2.55)	0.37		
≥25 nmol/L						
1 year	116.9 (22.2) (2866)	116.1 (21.7) (2854)	0.65 (−0.51, 1.81)	0.27	0.23	
2 years	123.5 (23.2) (2577)	123.0 (22.2) (2587)	0.48 (−0.71, 1.67)	0.43		
3 years	131.4 (26.2) (2743)	130.4 (25.3) (2697)	0.85 (−0.33, 2.02)	0.16		
By calcium intake						
<500 mg/day						
1 year	117.7 (22.0) (1461)	117.3 (21.9) (1412)	0.34 (−0.90, 1.59)	0.59	0.42	0.84
2 years	124.3 (23.3) (1355)	123.8 (22.6) (1301)	0.55 (−0.72, 1.81)	0.40		
3 years	131.8 (26.2) (1422)	131.1 (25.2) (1382)	0.65 (−0.60, 1.89)	0.31		
≥500 mg/day						
1 year	117.5 (22.8) (2562)	116.7 (21.9) (2579)	0.96 (−0.76, 2.67)	0.28	0.49	
2 years	124.0 (23.9) (2329)	124.3 (23.1) (2385)	0.31 (−1.44, 2.05)	0.73		
3 years	132.5 (27.0) (2510)	131.7 (25.7) (2527)	1.30 (−0.42, 3.03)	0.14		

* Adjusted for baseline value and school of attendance.

† After correction for multiple comparisons testing using the Benjamini and Hochberg method and a false discovery rate of 10%, this P-p value for interaction did not remain significant (critical threshold of significance for family of P-p values was <0.03).

‡ Deseasonalised values.

Nnumber25(OH)D25-hydroxyvitamin D

**Table 4:** Mean VO_2peak_ in exercise substudy participants at 1, 2 and 3 years follow-up by allocation: overall and by subgroup

	Vitamin D arm: mean VO_2peak_, ml/kg/min (SD) (n)	Placebo arm: mean VO_2peak_, ml/kg/min (SD) (n)	Adjusted mean difference (95% CI)*	P for time point	Overall p value	P for interaction
Overall						
1 year	43.2 (3.7) (278)	43.0 (3.4) (296)	0.27 (−0.30, 0.84)	0.36	0.28	--
2 years	41.9 (3.9) (268)	41.9 (3.7) (289)	0.20 (−0.37, 0.78)	0.48		
3 years	41.3 (4.2) (268)	41.3 (4.1) (286)	0.05 (−0.53, 0.62)	0.87		
By sex						
Male						
1 year	45.0 (3.1) (151)	44.5 (3.1) (144)	0.61 (−0.09, 1.32)	0.09	0.06	0.06
2 years	43.9 (3.1) (144)	43.5 (3.3) (143)	0.55 (−0.17, 1.26)	0.13		
3 years	43.6 (3.0) (143)	43.2 (3.8) (143)	0.46 (−0.25, 1.18)	0.20		
Female						
1 year	41.1 (3.3) (127)	41.6 (3.1) (152)	−0.32 (−1.05, 0.40)	0.38	0.47	
2 years	39.7 (3.5) (124)	40.2 (3.4) (146)	−0.40 (−1.13, 0.32)	0.28		
3 years	38.7 (3.8) (125)	39.4 (3.5) (143)	−0.68 (−1.41, 0.04)	0.07		
By baseline 25(OH)D concentration†						
<25 nmol/L						
1 year	43.0 (4.2) (103)	43.0 (3.2) (113)	−0.11 (−1.05, 0.83)	0.82	0.44	0.06
2 years	41.3 (4.0) (104)	41.9 (3.7) (107)	−0.62 (−1.56, 0.32)	0.20		
3 years	40.8 (4.6) (100)	41.3 (4.1) (108)	−0.64 (−1.59, 0.30)	0.18		
≥25 nmol/L						
1 year	43.4 (3.5) (175)	43.0 (3.5) (183)	0.50 (−0.22, 1.22)	0.18	0.050	
2 years	42.4 (3.7) (164)	41.8 (3.7) (182)	0.71 (−0.02, 1.43)	0.06		
3 years	41.6 (3.9)	41.3 (4.1) (178)	0.46 (−0.26, 1.19)	0.21		
By calcium intake						
<500 mg/day						
1 year	43.2 (3.9) (98)	42.8 (3.5) (102)	0.36 (−0.41, 1.14)	0.36	0.18	0.60
2 years	42.0 (4.1) (99)	41.5 (3.7) (102)	0.48 (−0.30, 1.25)	0.23		
3 years	41.2 (4.4) (96)	41.2 (4.2) (102)	0.13 (−0.64, 0.91)	0.74		
≥500 mg/day						
1 year	43.5 (3.4) (169)	43.3 (3.5) (174)	0.27 (−0.68, 1.22)	0.58	0.80	
2 years	42.1 (3.4) (161)	42.4 (3.7) (179)	0.01 (−0.94, 0.96)	0.99		
3 years	41.5 (3.9) (167)	41.5 (4.0) (176)	0.08 (−0.88, 1.03)	0.88		

* Adjusted for baseline value and school of attendance.

† Deseasonalised values.

Nnumber25(OH)D25-hydroxyvitamin DVO_2peak_peak oxygen consumption

**Table 5:** Spirometric outcomes at 3-year follow-up by allocation: overall and by subgroup

	Vitamin D arm: mean value (SD) (n)	Placebo arm: mean value (SD) (n)	Adjusted mean difference (95% CI)*	P value	P for interaction
**% predicted FEV1**					
Overall	101.5 (11.1) (666)	100.8 (11.0) (679)	0.75 (−0.44, 1.93)	0.22	--
By sex					
Male	99.5 (10.2) (333)	100.0 (10.9) (354)	−0.45 (−2.05, 1.16)	0.58	0.04†
Female	103.4 (11.5) (333)	101.7 (11.1) (325)	2.06 (0.31, 3.81)	0.021	
By baseline 25(OH)D concentration‡					
<25 nmol/L	102.0 (10.7) (232)	101.0 (11.3) (253)	1.31 (−0.67, 3.29)	0.20	0.71
≥25 nmol/L	101.2 (11.3) (434)	100.7 (10.8) (426)	0.63 (−0.86, 2.12)	0.41	
By calcium intake					
<500 mg/day	101.2 (10.9) (223)	100.7 (10.7) (244)	0.73 (−0.73, 2.19)	0.33	0.96
≥500 mg/day	102.1 (11.5) (435)	101.1 (11.6) (425)	0.88 (−1.26, 3.01)	0.42	
**% predicted FVC**					
Overall	101.6 (11.2) (666)	101.3 (11.5) (677)	0.34 (−0.88, 1.55)	0.59	--
By sex					
Male	100.2 (10.0) (333)	101.5 (10.9) (352)	−1.13 (−2.71, 0.46)	0.16	0.017
Female	103.0 (12.1) (333)	101.2 (12.1) (325)	1.82 (−0.05, 3.70)	0.06	
By baseline 25(OH)D concentration‡					
<25 nmol/L	102.2 (11.5) (232)	101.0 (11.9) (253)	1.36 (−0.75, 3.46)	0.21	0.34
≥25 nmol/L	101.3 (11.1) (434)	101.5 (11.2) (424)	−0.07 (−1.57, 1.43)	0.92	
By calcium intake					
<500 mg/day	101.5 (11.2) (223)	101.4 (11.4) (244)	0.23 (−1.30, 1.77)	0.77	0.87
≥500 mg/day	101.6 (11.3) (435)	101.1 (11.6) (423)	0.57 (−1.53, 2.68)	0.59	
**% predicted FEV1/FVC**					
Overall	98.0 (6.3) (666)	97.6 (6.4) (679)	0.49 (−0.19, 1.17)	0.16	--
By sex					
Male	97.0 (6.3) (333)	96.2 (6.6) (353)	0.72 (−0.25, 1.69)	0.15	0.49
Female	99.1 (6.2) (333)	99.0 (6.0) (326)	0.25 (−0.68, 1.18)	0.60	
By baseline 25(OH)D concentration‡					
<25 nmol/L	97.9 (6.2) (232)	98.4 (6.4) (254)	−0.27 (−1.40, 0.85)	0.64	0.08
≥25 nmol/L	98.1 (6.4) (434)	97.1 (6.4) (425)	0.96 (0.10, 1.82)	0.028	
By calcium intake					
<500 mg/day	97.8 (6.4) (223)	97.4 (6.6) (244)	0.51 (−0.37, 1.38)	0.26	0.99
≥500 mg/day	98.5 (6.3) (435)	98.0 (6.0) (425)	0.45 (−0.68, 1.58)	0.43	
**% predicted PEFR**					
Overall	95.2 (14.1) (666)	93.6 (13.6) (680)	1.69 (0.21, 3.18)	0.026	--
By sex					
Male	94.2 (13.4) (333)	93.1 (14.0) (354)	1.08 (−0.98, 3.13)	0.30	0.33
Female	96.3 (14.8) (333)	94.1 (13.2) (326)	2.42 (0.24, 4.59)	0.030	
By baseline 25(OH)D concentration‡					
<25 nmol/L	96.0 (13.4) (232)	93.5 (13.9) (254)	2.67 (0.19, 5.15)	0.035	0.47
≥25 nmol/L	94.8 (14.5) (434)	93.6 (13.5) (426)	1.30 (−0.58, 3.18)	0.18	
By calcium intake					
<500 mg/day	94.3 (13.9) (223)	92.8 (13.2) (244)	1.76 (0.93, 3.59)	0.060	0.97
≥500 mg/day	97.1 (14.7) (435)	95.1 (14.2) (426)	1.47 (−1.18, 4.12)	0.28	
**% predicted FEF25–75**					
Overall	102.4 (21.9) (667)	100.7 (21.8) (680)	1.47 (1.19, 3.82)	0.22	--
By sex					
Male	99.8 (21.2) (334)	98.0 (22.2) (354)	1.17 (−2.14, 4.48)	0.49	0.73
Female	105.0 (22.3) (333)	103.6 (20.9) (326)	2.11 (−1.20, 5.42)	0.21	
By baseline 25(OH)D concentration‡					
<25 nmol/L	102.0 (21.6) (232)	101.4 (21.7) (254)	1.19 (−2.69, 5.08)	0.55	0.79
≥25 nmol/L	102.7 (22.1) (435)	100.3 (21.8) (426)	1.84 (−1.14, 4.81)	0.23	
By calcium intake					
<500 mg/day	100.9 (20.2) (223)	99.9 (21.8) (244)	0.92 (−1.94, 3.77)	0.53	0.55
≥500 mg/day	105.6 (24.9) (436)	102.5 (21.7) (426)	2.38 (−1.88, 6.65)	0.27	

* Adjusted for school of attendance.

† After correction for multiple comparisons testing using the Benjamini and Hochberg method and a false discovery rate of 10%, this P-p value for interaction did not remain significant (critical threshold of significance for family of P-p values was <0.007).

‡ Deseasonalised values.

FEF25–75forced mid-expiratory flowFEV1forced expiratory volume in 1 sFVCforced vital capacityNnumber25(OH)D25-hydroxyvitamin DPEFRpeak expiratory flow rate

## Discussion

We present findings of the largest RCT to investigate the effects of vitamin D on muscle strength, peak oxygen uptake and spirometric lung volumes in children. Vitamin D deficiency was highly prevalent among the study population at baseline, and the intervention was highly effective in elevating serum 25(OH)D concentrations among participants who were randomised to receive it. However, this was not associated with any effect on any physiological outcome investigated, either overall or in subgroups defined by baseline vitamin D status, sex or calcium intake.

Our findings contrast with those of observational studies reporting associations between low vitamin D status and reduced muscle strength and cardiorespiratory fitness,^12 13^ and chime with results of smaller RCTs, conducted in populations with lower prevalence of vitamin D deficiency, that have yielded null results.^18^,^22^ They are also consistent with the lack of effect seen for muscle strength and exercise outcomes in a similar trial conducted in South Africa,^38^ and for other ‘non-classical’ outcomes in trials of weekly vitamin D supplementation in children.^23^,^2539^ Inconsistency between positive findings of observational versus null findings from interventional studies may reflect effects of confounding or bias in the former.^42^ Lack of subgroup effects in participants with lower baseline vitamin D status or calcium intake suggests that neither of these factors modified the effects of vitamin D supplementation on outcomes investigated. We highlight that our null findings do not have relevance for children with symptomatic vitamin D deficiency since those who were found to have signs of rickets were excluded from the trial, as it would not have been ethical to randomise them to placebo.

Our study has several strengths. The large sample size and low rates of loss to follow-up maximised our power to detect the effects of the intervention, and the high prevalence of vitamin D deficiency and low calcium intake at baseline allowed us to rule out effects even in subgroups who might have been expected to derive particular benefit from vitamin D replacement. The intervention was highly effective in elevating serum 25(OH)D concentrations into the physiological range. We also assessed a comprehensive range of outcomes relating to muscle and cardiorespiratory fitness, with assessments at more than one time point to allow detection of any effects that may have differed with varying duration of supplementation.

Our study also has some limitations. Spirometry was assessed at a 3-year follow-up only: accordingly, analyses testing the effect of allocation to vitamin D versus placebo could not be adjusted for baseline. However, we have no reason to suspect a significance imbalance in baseline values between groups, as the randomisation process was effective in distributing all other baseline characteristics evenly between children randomised to intervention versus control arms. We acknowledge that the outcomes investigated in the current study were secondary, rather than primary, outcomes (although prespecified ones), and that multiple tests for statistical significance were performed. Statistically significant findings may, therefore, have arisen as a result of type 1 error. We addressed this issue by applying a correction for multiple comparison testing. We also highlight that our findings relate to effects of weekly vitamin D supplementation specifically. It remains technically possible that daily supplementation might have a different effect, although the fact that weekly supplementation is effective in suppressing serum concentrations of parathyroid hormone and alkaline phosphatase in study participants^25 40^ suggests that this dosing regimen exerts the same physiological effects as would be expected with daily supplementation. Further trials comparing effects of daily versus weekly supplementation would be needed to resolve this question definitively.

In conclusion, this large multicentre RCT of vitamin D supplementation, administered for 3 years to a population of children with low baseline 25(OH)D concentrations, did not show any effect of the intervention on muscle strength, cardiorespiratory fitness or spirometric outcomes. Taken together with null results from a similarly designed phase 3 RCT conducted in CapeTown, South Africa,^38^ our study does not suggest a role for weekly oral vitamin D supplementation to enhance muscle strength, peak oxygen uptake or respiratory function in schoolchildren in whom rickets has been excluded.

## supplementary material

10.1136/bmjsem-2024-002018 online supplemental file 1

## Data Availability

Data are available on reasonable request.
