# Supplementary material for: Influence of vitamin D supplementation on muscle strength and exercise capacity in Mongolian schoolchildren: secondary outcomes from a randomised controlled trial
Source: BMJ Open Sport Exerc Med. 2024 Sep 26;10(3):e002018. doi: 10.1136/bmjsem-2024-002018 (PMC11429264; doi:10.1136/bmjsem-2024-002018)
Supplement: online supplemental file 1 [file bmjsem-10-3-s001.pdf]

Protocol

This trial protocol has been provided by the authors to give readers additional information about their work.

Protocol for: Ganmaa D, Uyanga B, Zhou X, et al. Vitamin D supplements for prevention of tuberculosis infection and disease. N Engl J Med 2020;383:359-68. DOI: 10.1056/NEJMoa1915176

Protocol Supplement

This supplement has been provided by the authors to provide readers with original and final versions of the protocol, as well as a summary of the changes made to the original version.

Supplement to:

Ganmaa D, Uyanga B, Zhou X, et al. Vitamin D Supplementation to Prevent Tuberculosis Infection and Disease.

Vitamin D Supplementation to Prevent Tuberculosis Infection and Disease

PROTOCOL SUPPLEMENT

Contents

1.Original Version of the Protocol/SAP ………………………………………………......3

2.Final Version of the Protocol/SAP ………………………………………………… 21

3.Summary of Changes to the Protocol/SAP……………………………………….....49

1. Original Version of the Protocol

GENERAL INFORMATION	
**Protocol #** (if assigned): **14-0513**	
**Version Date: 2/12/2015**	**Version Number: v1.0**
**Principal Investigator: Davaasambuu Ganmaa**	
**Faculty Advisor (**if PI is a student**):**	
**Protocol Title: Vitamin D in TB Prevention in School Age Children**	

1. **Specific Aims**

   Primary aim. Determine the extent to which 14000 IU of vitamin D weekly (equivalent to 2000 IU daily) reduces the risk of latent tuberculosis infection (LTBI) in children.
   Secondary Aim 2. Determine whether this regimen of supplementation reduces incidence of active TB disease.
   Secondary Aim 3. Determine whether any protective effect of vitamin D supplementation is dependent on baseline serum 25-hydroxyvitamin D (25[OH]D) concentration.

2. **Background**

   2.1. Provide the scientific background and rationale for the study

   Tuberculosis (TB) is an infectious disease caused by the bacterium *Mycobacterium tuberculosis* (*M. tuberculosis*) and a major global cause of morbidity and mortality; the World Health Organization (WHO) estimates that 8.7 million cases caused 1.4 million deaths worldwide in 2011. Almost all TB cases (94%) and deaths (98%) occur in developing countries, and 75% of cases arise among people in the economically productive age group (15-54 years). Mongolia has one of the largest numbers of incident TB cases in the world (16). The registered incidence of TB in Mongolia has increased from 79 to 141 per 100,000 between 1990 and 2001, and WHO estimates incidence at 230 per 100,000. Moreover, latent tuberculosis infection (LTBI) is most commonly acquired in childhood, and the rates of acquisition of LTBI in children in Mongolia are particularly high – 2% per annum (12).

   Both in vitro and animal studies provide evidence of a protective relationship between vitamin D and the risk of acquiring latent TB infection. The evidence underpinning the trial we propose is compelling. For instance, twenty-five years of in vitro studies have consistently found that vitamin D metabolites induce innate immunity to mycobacteria in vitro via pleiotropic mechanisms, including induction of reactive nitrogen intermediates, reactive oxygen intermediates, antimicrobial peptides and autophagy (10) as first suggested by Rook (17) and Crowle (18). An in vitro study with human lung macrophages found that interferon gamma (IFN-γ) enhanced the killing activity of macrophages against *M. tuberculosis* in a dose-dependent manner, and that 1,25-D3, the biologically active form of vitamin D, synergistically augmented the effect of IFN-γ (19). More recently, Liu et al. showed that 25(OH)D supports TLR ligand-induced expression of the antimicrobial peptide cathelicidin and restriction of growth of intracellular *M. tuberculosis* (20). TLR induction led to cathelicidin activity, except when either the vitamin D receptor or the vitamin D activating enzymes were inhibited. Vitamin D deficiency has also been associated in vivo with increased progression of TB among infected mice and in cattle infected with *M. bovis* (21-23).

   Human studies indicate that vitamin D deficiency may increase TB susceptibility (24,25). A systematic review of case-control studies found consistent independent associations between vitamin D deficiency and susceptibility to TB (9). Serum 25(OH)D was found to be lower in untreated TB patients than in healthy controls in the UK (26) and to be associated with disease severity in Indonesia (27). Gibney (28) reported an association between higher vitamin D

status and negative tuberculin status in asymptomatic people. Arnedo-Pena (29) showed that sufficient serum 25(OH)D concentrations are inversely associated with skin test conversion in a cohort study of people recently exposed to an infectious case. Despite substantial declines in TB in the United States, blacks continue to have TB at rates eight times greater than whites (30). Blacks also have virtually half the levels of 25(OH)D compared to whites, presumably due to lower synthesis of vitamin D in skin with greater melanin content, which blocks UV-B penetration. In racially integrated nursing homes, infection measured by tuberculin skin test conversion occurred twice as often in black as in white individuals who were equally exposed to active TB (31). In accordance with this, macrophages from blacks demonstrate a relative permissiveness for intracellular growth of virulent mycobacteria (32). A systematic review of observational studies reports that the association between vitamin D deficiency and susceptibility to TB is strong and widely replicated, and concluded that trials of vitamin D supplementation for TB prevention should be conducted.

2.2. Describe the significance of the research, and how it will add to existing knowledge

Two Phase 2 randomized placebo-controlled clinical trials have reported that in vivo vitamin D supplementation enhances antimycobacterial immunity. The first, conducted in London by our collaborator, Dr Martineau, found that bolus-dose vitamin D supplementation taken by healthy people recently exposed to infectious TB cases enhanced the ability to restrict mycobacterial growth ex vivo by 25%(11). The second, conducted by our team in schoolchildren living in Mongolia (12), showed that vitamin D supplementation resulted in a 59% reduction in risk of tuberculin skin test (TST) conversion. While this finding suggests that vitamin D supplementation does indeed enhance innate immune resistance to LTBI, its interpretation is complicated by the fact that repeated tuberculin skin testing may lead to ‘false positive’ skin test conversions in the absence of LTBI (the boosting phenomenon). A more sensitive and specific means of diagnosing LTBI than the TST is to use interferon-gamma release assays (IGRA), which are not subject to the boosting phenomenon (33).

We therefore consider this the appropriate time to conduct a definitive Phase 3 clinical trial to determine whether vitamin D supplementation can prevent IGRA-diagnosed LTBI in populations living in high TB-transmission settings where vitamin D deficiency is very common. This research is significant, as positive results would mean an inexpensive, non-toxic means of reducing the incidence of a major global killer. The impact of this work will be particularly significant for children who live in high-risk TB settings, given that diagnoses of TB in children are difficult and children are at much greater risk of developing dangerous forms of disseminated disease such as military, or CNS, TB. In addition to their public health impact, the results will also inform clinical decision-making at the individual level, as the data will provide clinicians with an evidence base to inform their management of vitamin D-deficient patients who are at risk of TB. Impact from our research work could help in the design of preventive agents that would benefit millions of individuals and limit the spread of *M. tuberculosis* worldwide.

3. Study Setting

3.1. Identify the sites or locations where the research will be conducted.

Mongolia provides an excellent setting in which to conduct a study of this nature; vitamin D levels are low, supplementation is rare, the prevalence of TB is high, HIV co-infection is extremely low, the health care system is organized, participants are well educated and have enrolled in our studies in high numbers.

There are many reasons why 25(OH)D levels are low in Mongolia. Ulaanbaatar is located at a latitude of 48°N where UVB exposure from October through April is weak and temperatures preclude skin exposure. Furthermore, Mongolians do not typically consume vitamin D rich foods, their food supply is not fortified with vitamin D, and few people use vitamin D supplements.

3.2. Describe the Principal Investigator's experience conducting research at study site(s) and familiarity with local culture

Dr. Ganmaa has conducted several studies in Mongolia, including a six-armed randomized trial of 744 participants, and 420 premenopausal women simultaneously in 2009. As a co-investigator in another randomized, placebo controlled trial, she showed that vitamin D in a dose of 1000IU resulted in a modest, but statistically significant reduction in winter-related eczema in Mongolian children. As PI, Dr. Ganmaa completed a randomized clinical feasibility trail among Mongolian school-age children to test effect of vitamin D supplementation against latent TB infection. Currently, she is running three active studies on vitamin D in Mongolia. As a native Mongolian, she is familiar with the culture and system and thus she was able successfully administer the research projects.

3.3. Is the research conducted outside the United States?

☒Yes: If yes; describe site-specific regulations or customs affecting the research, local scientific and ethical review structure

The proposal will be reviewed by the Institutional Review Board at Harvard School of Public Health, the Ethical Committee of the Ministry of Health in Mongolia, the Ethical Committee of Health Sciences University of Mongolia, and the Institutional Review Board of National University of Mongolia, FWA00012628.

3.4 Are there any permissions that have been or will be obtained from cooperating institutions, community leaders, or individuals, including approval of an IRB or research ethics committee? ☒Yes: If yes; provide a list of the permissions (also include copies with the application, if available)

We will apply for the Mongolian Ministry of Health IRB, , and National University of Mongolia IRB. As with our previous protocols in Mongolia, the ERB prefer for us to first receive HSPH approval before submitting for their review.

4. Study Design

4.1. Describe the study design type

We propose conducting a placebo-controlled, double-blind randomized trial of vitamin D to prevent TB infection among school-aged children in Ulaanbaatar, the capital of Mongolia.

4.2. Indicate the study's duration - and the estimated date of study completion

The study duration is five years with intervention running for three years. The estimated date of study completion is December 2019.

4.3. Indicate the total number of participants (if applicable, distinguish between the number of participants who are expected to be screened and enrolled, and the number of enrolled participants needed)

Approximately 8,220 participants will be recruited for the trial.

4.4. List inclusion criteria

In order to maximize generalizability and representativeness, we deliberately set inclusion criteria to be broad. Eligible subjects are boys and girls without TB infection, ages 6 to 12 years inclusive, who are residents of Ulaanbaatar, who have given informed consent to participate in the study, whose parents have given informed consent, and who assent to participate in the study. Those testing positive for LTBI on screening will be assessed for active TB and referred for treatment. Children with clinical signs of rickets will be assessed by a study doctor and treated with vitamin D and calcium supplements.

4.5. List exclusion criteria

As necessitated by the study design, the presence of latent tuberculosis infection (LTBI) at screening will result in exclusion from the study. Children positive for LTBI on screening will be assessed for active TB and referred for treatment. Due to the high prevalence of TB in Mongolia, it will be not stigmatizing for those children who are ineligible due to positive LTBI.

Children with rickets at screening will also be excluded as to avoid randomizing those children to the placebo group. Additionally the followings will be excluded:

• Known HIV sero-positivity, primary hyperparathyroidism or sarcoidosis at screening

• Taking immunosuppressant or cytotoxic therapy, vitamin D supplement > 10 micrograms / day, or 1-alpha-hydroxylated vitamin D preparations

Plans to move away from study area within 3 years of enrollment

4.6. Describe study procedures

Screening procedures and baseline data: Assenting children whose parents also give consent for them to participate will be screened for eligibility to participate by trial staff. Those fulfilling all eligibility criteria will be allotted a unique interim ID code, consisting of a 2-digit ‘school code’ from 01 to 22 (unique to each school), followed by a 3-digit ‘participant code’ from 001 to 400 (unique to each participant at that school, assigned consecutively in order of enrolment), followed by a four letter code. Each part of this interim ID code will be separated by a dash (e.g. 01-001-GADA, 01-002-BAGU, etc). Children will be physically examined at baseline by study doctors for clinical signs of rickets, will then undergo a baseline assessment, anthropometry, grip strength, whole body impedance and interviewed by research fellows detailing demographic and clinical data, including the ISAAC questionnaires for asthma, eczema and allergic rhinitis. Physical fitness will be determined using the 20 metre multistage shuttle test (34) in a n=420 sub-sample of participants. BMD will also be determined using quantitative ultrasound bone densitometry in a separate n=1200 sub-sample of participants. At this point, baseline blood samples will be taken for vitamin D status, which will be determined in stored samples for all participants at the end of the trial. Participants will be screened for presence of LTBI at baseline, using a validated commercial whole blood assay, the QuantiFERON-TB Gold in-tube assay (QFT-G) and whole blood samples will be stored in deep freezer. The Mongolian National University of Medical Sciences (MNUMS) and National Center for Communicable Disease (NCCD) lab will provide a comprehensive package incorporating these services. When the result of the QFT-G test is available, participants with a negative QFT-G result will be randomized as detailed below, and invited to attend a second study visit for administration of first dose of vitamin D/placebo. Those with a positive QFT-G result will not be randomized, but they will also be invited to attend a second study visit to undergo screening for active TB

Randomization: Participants will be randomized to receive vitamin D or placebo in a 1:1 ratio; randomization will be stratified by school. We will also stratify randomization by school. Randomization is stratified by school because school of attendance may be a strong risk factor for LTBI acquisition and we want to ensure that treatment assignment is balanced within this risk factor to avoid confounding in treatment effect estimation due to random imbalances. If a TB outbreak occurs in a particular school, children attending it might be at increased risk of acquiring LTBI. Prior to the start of recruitment, a statistician who is independent of the trial will prepare one school randomization.

The school randomization list will comprise 22 paired 2-letter randomization codes (one pair of randomization codes per school, e.g. School 1: AA/AB, School 2: AC/AD etc). One randomization code within each pair will be randomly assigned to the active arm of the trial, and the other will be assigned to the placebo arm, using a computer-generated random sequence. The resulting school randomization list will be sent to Investigational Drug Services (IDS) at BWH; copies will be held by members of the DSMB, and by a statistician

Prof. Chadraa who is independent of the trial, but participants and trial staff will not have access to it. The statistician who is independent of the trial will access this list in the event that a code break is required.

Preparation and use of participant randomization lists:
Each of the 22 participant randomization lists will initially comprise 400 5-digit numeric codes corresponding to participants' unique interim ID codes, i.e. consisting of a 2-digit school identifier from 01 to 22, followed by a 3-digit participant identifier from 001 to 400, separated by a dash (e.g. 01-001, 01-002). These 5-digit codes will then be randomly assigned to one or other of the 2-letter randomization codes in use for the corresponding school in blocks of ten, using a computer-generated random sequence. The resulting participant randomization lists will be supplied to the trial team, who will use them to append 2-letter randomization codes onto the end of eligible, randomized participants' interim ID codes, separated by a dash, to create a final ID code for each (e.g. if the participant randomization list indicates that participant 01-001 has been assigned to randomization code AA, participant 01-001-GADA's interim ID code will become final ID code 01-001-GADA-AA; similarly, if the participant randomization list indicates that 01-002 has been assigned to code AB, participant 01-002-BAGU's interim ID code will become final ID code 01-002-BAGU-AB). Participants who are ineligible for randomization will have the letters XX appended onto the end of their interim ID code to generate their final ID code. The enrolment of randomized participants will be documented in an enrolment log.
Intervention: The intervention will run over three school years. Parents will be asked to give study capsules during the summer and school holidays, and compliance will be monitored by counting the number of empty bottles returned. The content of the vitamin D and placebo will be calibrated in advance by Tishcon Inc. One capsule of 14000 IU D3 will be given weekly on schooldays. A field worker will be hired, trained, and assigned to each school as a 'vitamin D monitor.' These monitors will receive vitamin D/placebo capsules from study staff, and distribute them accordingly to participating children in a private room in their school. The monitors will observe that the vitamin D/placebo is consumed and will record any noncompliance. The project coordinator and a research fellow will supervise and retrain the 'vitamin D monitors' at regular intervals to ensure safety and maintain quality control.

Study Visits (see Table 1): Both the treatment and follow-up periods will be three years from enrolment. Administration of all doses of study medication will be directly observed by the study teams to optimize compliance. Height, weight, ISAAC questionnaires,-body impedance, grip strength and bone mineral density (BMD) (in subsets of the trial population), physical fitness, cognitive test and BMI for age will be determined at annual follow-up using validated scales and the portable height measure (UNICEF supply catalogue S0141021); BMI will be calculated as weight (kg)/[height (m)]^2. Questionnaires will be entered using REDCap secure database by trained research fellows. Weekly administration of vitamin D and placebo, recording of Serious Adverse Events (SAE) will be performed on an on-going basis throughout the duration of the trial. Those with symptoms of active TB at monthly follow-up (i.e. fever and/or cough lasting ≥2 weeks, or weight loss >5% of the highest weight recorded in the previous 3 months) and those with history of recent household contact with an infectious TB case will be examined by a study doctor for signs of active TB using WHO criteria.

Table 1. Study visits

Visit number	1	2	3	4	5	6	7	8	9	10	11	12	13	14
Visit timing	-1	0	3	6	9	12	15	18	21	24	27	30	33	36
Informed consent, assent	X													
Check eligibility criteria	X													
Record baseline data	X													

QFT-G	X													X
25(OH)D	X													X
Storage of whole blood	X													
Measure height	X					X				X				X
Measure weight	X		X	X	X	X	X	X	X	X	X	X	X	X
Measure whole-body impedance	X					X				X				X
Measure grip strength	X					X				X				X
Fitness test (multistage shuttle test; n=420 subset)	X					X				X				X
Cognitive test	X					X				X				X
ISAAC questionnaires	X					X				X				X
BMD (n=1200 subset)	X					X				X				X
Randomization		X												
First dose of vitamin D		X												

1. Performed monthly 2. Performed weekly; study medication to be administered by parents during summer vacations.

4.7. Does the study involve the collection of data/specimens (including the use of existing data/specimens)?

☐No ☒Yes: If yes; indicate how, when, where and from whom specimens or data will be obtained

Blood samples at baseline and at the end of the three-year follow-up will be taken in the morning at the school clinic by experienced phlebotomists to assess 25(OH)-vitamin D levels. Serum 25(OH)D concentration will be determined by Diasorin/Liason in batches at the end of the trial in stored serum from all participants. QFT-G test will be performed at baseline and at 3 years post-randomization.

4.8. Is there a data and safety-monitoring plan (required for greater than minimal risk studies)?

☐No ☒Yes: If yes; describe the plan

A data and safety monitoring board (DSMB) will be formed and will review interim safety and efficacy analyses at 12-month intervals. The DSMB will include independent U.S. and Mongolian scientists, statisticians, and clinicians with relevant experience. The DSMB will have the authority to stop any intervention arm if they determine that it is having an adverse impact on participant health. Trained data entry personnel will collate and clean the data detailing all known serious adverse events to date in trial participants, and the study statistician, Dr. Spiegelman, will produce reports in accordance with the requests of the DSMB. Each meeting will include an administrative review to assess accrual, retention, and the progress of the study. In addition, there will be a blinded review of safety. A single efficacy analysis will be conducted on completion of the trial; no interim efficacy analyses will be conducted as data relating to the primary outcome (i.e. QFT-G test results) will not be available until the end of the study. Fatal and life-threatening significant adverse events (SAE) will be defined by the DSMB in consultation with the Trial Steering Committee. The DSMB will decide whether or not to unblind the study, and whether or not to consult with the sponsor and IRBs to decide whether the trial should be stopped, taking into account the severity of the SAE, biological plausibility, consistency with other study data, and the strength of the association.

DSMB Schedule: We propose that the DSMB meetings by conference call to decide the “stopping rule” protocol. A detailed Data and Safety Monitoring Plan will be submitted to

the ERB and the Harvard Human Subjects Committee for approval prior to the recruitment of participants. The study biostatistician and PI will prepare reports for the DSMB, including data on participant recruitment, retention, compliance, and adverse events. The DSMB will meet at least annually during the course of the study. We propose the following members of the DSMB:

- Professor Holick (expert on vitamin D, Boston University).
- Dr. James Ware (biostatistician from the Harvard School of Public Health) with extensive experience in DSMBs for clinical trials
- Dr. Sarah Fortune (Associate Professor of Immunology and Infectious Diseases, Harvard School of Public Health, TB expert the State Research Center on Maternal and Child Health, Mongolia).
- Prof. Perenlei Enkhbaatar (University of Texas Medical Branch, Galveston, TX) has 12 years of clinical and teaching experience in lung disease both in Mongolia and the US.
- Dr. Erdenebaatar Chadraa (Assistant Professor of Mathematics, Minnesota State University).

In addition to the above measures, we will also convene a Scientific Advisory Board to advise the study. We will seek their input in evaluating risks and benefits, as well as the most effective ways to communicate risks and benefits (and eventually, study findings) to individuals and institutions participating in the study and to the public at large.

4.9. Are there any anticipated circumstances under which participants will be withdrawn from the research without their consent?

☒No ☐Yes: If yes; describe the circumstances as well any associated procedures to ensure orderly termination

5. Data/Statistical Analyses Plan

5.1. Briefly describe the plan for data analysis (including the statistical method if applicable)

To compare acquisition of LTBI in the intervention vs. control arms during the three-year follow-up, we will perform an intent-to-treat analysis. Differences in the proportion of children who acquire LTBI during the three-year period who were randomized to placebo will be compared to those randomized to the vitamin D regimen, using the Mantel-Haenszel risk ratio (51), stratified by school.

To test the statistical significance of any difference observed, the Mantel-Haenszel test will be used. No adjustment is needed for multiple comparisons or interim monitoring as the endpoint will be assessed only at the three-year end of study period. Binary safety endpoints will be analyzed similarly; the significance of differences observed in continuous safety endpoints between the two arms will be assessed through the robust Wald test in a linear model with a binary indicator for treatment assignment, and a binary indicator for all of the schools except one.

Time to diagnosis of active TB disease

Assuming that the assumption of proportional hazards is not violated, time to first diagnosis of active TB will be compared between intervention vs. control arms using Cox regression allowing for a shared frailty within the same school, with frailty following a gamma distribution. The adjusted hazard ratio will be presented with 95% confidence interval and P-value.

Time to first acute respiratory infection / asthma exacerbation resulting in hospitalization

Analyses will use Cox regression as above.

BMI, whole-body impedance, grip strength, BMD (in n=1,200 sub-set only) at 1, 2 and 3 years post-randomization

Analyses will use linear regression with nested random effects of school and individual, constrained so that there is no treatment effect at baseline, and with a treatment effect estimated at each subsequent time-point. Outcomes with highly skewed distributions may be transformed prior to analysis. Adjusted treatment mean differences at different time-points will be presented with 95% confidence intervals. A P-value for treatment-time interaction will be used to evaluate evidence for an effect of treatment, and if evidence is found (P<0.05) then the P-values for the treatment effect at individual time-points will be reported.

Physical fitness at 1, 2 and 3 years post-randomization

Number of laps completed in the shuttle run test will be used to estimate VO2max using published formulae. (38) The effect of allocation on mean VO2max will then be analysed using linear regression as described above.

Incidence of asthma, eczema and allergic rhinitis as indicated by ISAAC questionnaire performed at 1, 2 and 3 years post-randomization

Analyses will use logistic regression with nested random effects of school and individual, constrained so that there is no treatment effect at baseline, and with a treatment effect estimated at each subsequent time-point. Adjusted odds ratios for treatment at different time-points will be presented with 95% confidence intervals. A P-value for treatment-time interaction will be used to evaluate evidence for an effect of treatment, and if evidence is found (P<0.05) then the P-values for the treatment effect at individual time-points will be reported.

Cost-effectiveness of vitamin D supplementation for the prevention of LTBI / TB

We will conduct an economic evaluation to estimate the within-trial cost-effectiveness of using vitamin D to prevent LTBI. Rates of LTBI in the two study arms will be determined at 36 months post-randomization. . Based on trial data, a decision analytic model will be developed to estimate the cost-effectiveness of vitamin D supplementation in the prevention of LTBI in schoolchildren compared to usual practice. The cost of LTBI prevention will include costs of procuring, transporting, storing and administering vitamin D supplementation; costs of staff involved in vitamin D procurement and administration; and costs of producing information about vitamin D supplementation for parents/guardians. Any costs associated with serious adverse events will also be recorded

5.2. Is there a sample size/power calculation?

☐No ☒Yes: If yes; describe the calculation and the scientific rationale, and, if applicable, by site and key characteristics such as participant demographics

Styblo’s Rule (55) predicts that for every 50 incident smear positive cases per year per 100,000 population, one would expect a 1% increase in ARTI. WHO estimates the TB incidence in Mongolia at 230 per 100,000 per year. There is some disagreement about the current validity of Styblo's rule (56), particularly in areas of high HIV prevalence and low TB prevalence, but the Mongolian situation is opposite to that and more typical of TB epidemiology in the pre-HIV era: in Mongolia, the prevalence of TB is high, and HIV co-infection is extremely low (57). Using Styblo’s Rule, and based on a TB incidence rate as estimated by WHO of 230 cases per 100,000 in Mongolia we estimate the annual ARTI rate in our study population will be 2% (44% of the 230 cases per 100,000 per year are smear positive) (56), which leads to an estimate that 5.9% of our subjects will become infected over 3 years of follow up. If, thinking conservatively, we expect vitamin D supplementation to reduce that predicted rate by 25%, as we assumed in our initial proposal; we get a predicted infection rate in the intervention arm of the study of 4.4% over 3 years.

Assuming 10% loss to follow-up (based on interviews with head teachers at participating schools and on our previous studies in Mongolia) (58,59), and an additional 5% lost due to indeterminate results of the QFT-G at the end of the study (60-62), we would need 4,010 subjects per arm, for a total of 8,020 (rounding up to the nearest ten subjects) to detect the hypothesized treatment effect with 80% power.

6. **Recruitment Methods**

6.1. **Does the study involve the recruitment of participants?**

☐**No: If no, skip to <u>7.1</u>**

☒**Yes: If yes; indicate how, when, where, and by whom participants will be recruited**

There are 710 secondary schools in Ulaanbaatar, with a total of 557,346 children. Recruitment will take place over one year, in partnership with the Ministry of Education of Mongolia (MOEM) and the Government of Mongolia, which have responsibility over participating schools and have agreed to collaborate on this project (please refer to the enclosed letter). Participants will be recruited from twenty primary schools in Ulaanbaatar, selected on the basis of: lower socio-economic status (as these children are at potentially higher risk of acquiring LTBI and having baseline vitamin D deficiency); large size (as fewer schools will need to be visited in order to attain our target sample size, which reduces study costs); and proximity to the trial co-ordination center in Ulaanbaatar, reducing transport time and study costs. Based on an ARTI of 2% and results from feasibility trial (12), we estimate that 15% to 28% of 6-13 year olds will already have LTBI at screening, and will thus be ineligible for randomization; however, we will still be able to meet our target sample size.

Based on our experience of conducting clinical trials in children in Ulaanbaatar, and on interviews with school directors in participating schools, we estimate that no more than 10% of otherwise eligible participants and/or parents will decline to take part in the study. Thus, in an average primary school with 700 pupils in classes 1-7, we anticipate that we will be able to randomize 500 pupils. Based on these assumptions, fifteen schools would need to be screened, and to ensure that our recruitment targets are reached, we have inflated this number to twenty two schools.

6.2. **Are there any materials that will be used to recruit participants, e.g., emails, posters, and scripts?**

☐**No** ☒**Yes: If yes; provide a list of the materials (also include copies with the application)**

A letter will be sent home to parents explaining the study and inviting parents and children to come to twenty-five informational meetings about the study (thus keeping each meeting to 40 parent/child pairs). Literacy rates are quite high in Mongolia (95% of adults can read) meaning written communication is likely to be read and understood. (Please also see enclosed script.)

7. **Available Resources**

7.1. **Describe the feasibility of recruiting the required number of participants within the recruitment period**

In Mongolia, a research team of 22 field workers, supervised by 5 research fellows, will be responsible for recruiting approximately 10,225 participants, and ideally enrolling about 8,200 of those who are eligible for randomization. Additionally, they will perform monthly follow-up visits to the 22 participating schools, and pursue participants who do not attend these visits in order to minimize loss to follow-up. The field workers and research fellows will be supervised by a full-time trial coordinator and supported by a full-time project manager. Three study doctors will be responsible for assessing participants for development of active TB and fielding day-to-day medical queries from the study team. A team of four drivers will transport the study team to participating schools, and drive samples and participants requiring chest X-rays to the NCCD laboratory and clinical facilities in Ulaanbaatar.

7.2. **Describe how the Principal Investigator will ensure that a sufficient amount of time will be devoted to conducting and completing the research**

Dr. Ganmaa will have detailed oversight of day-to-day management of the trial in Mongolia and will coordinate applications for regulatory approval there. She will be assisted by a full-time trial coordinator, who will supervise a team of four research fellows and twenty fieldworkers responsible for recruitment and follow-up of study participants. A full-time project manager will also be employed to liaise with ethics and regulatory bodies, coordinate recruitment of the study team and handle administrative paperwork. On a daily basis, the project coordinator will communicate with the Boston office. Once weekly, Dr. Ganmaa, the project manager, coordinator, and local team will meet by Skype conference call. Dr. Ganmaa will oversee data collection/management; design, analyses, and travel to Mongolia; contribute to clinical interpretation of results; statistical analysis; and lead on write-up. She will also liaise with the study coordinator in Mongolia on a daily basis and the local staff and study doctors on a weekly basis, to perform study monitoring, and oversee laboratory services (NCCD lab) and data entry, to ensure that all study procedures comply with the sponsor-approved protocol, and will maintain oversight of study quality.

7.3. Are there research staff members, in addition to the Principal Investigator?

☐**No: If no, skip to** <u>**7.5**</u>

☒**Yes: If yes; outline training plans to ensure that research staff members are adequately informed about the protocol and study-related duties**

The Trial Steering Committee (TSC) with representation from investigators will be established with responsibility for overall management of the study. The committee will consist of Drs. Willett, Martineau, Bloom, Giovannucci, Spiegelman, Malchinkhuu, Naranbat and Ganmaa. They will lead on study design, provide expertise in nutrition trials, trial methodology, vitamin D and TB immunology and immunodiagnostics, and will contribute to clinical interpretation of results and write-up. Dr. Spiegelman will be the study's biostatistician, contributing statistical expertise and advising on study design and sample size calculations. She will prepare reports to the DSMB, write the statistical analysis plan, lead on statistical analysis, and contribute to write-up. Dr. Naranbat, former head of the NCCD TB Surveillance Division, will lead oversight and conduct of the trial in Mongolia.

Collaboration: Our newly-formed partnership with the Ministry of Education will be fostered by including a representative of the Ministry of Health, Trial Steering Committee (TSC), and by arranging 6-monthly meetings between Drs. Naranbat and Dr. Erkhembulgan, Deputy Director of Policy Implementation Department and Head of Education in Mongolian Ministry of Education (MMOE). Professor Malchinkhuu has longstanding links with both the Ministry of Health as a chief expert pediatrician of the MMOH and Pediatric Specialty Committee of Mongolia. He will keep these organizations informed about progress of the trial as it proceeds. Both organizations have contributed to the design of the research.

7.4. Describe the minimum qualifications for each research role (e.g., RN, social worker) their experience in conducting research, and their knowledge of local study sites and culture

Drs. Martineau, Willett, Bloom, and Giovannucci have many years of experience in forming and following large cohorts and randomized clinical trials, both in Boston and internationally. Dr. Martineau is currently conducting several trials of vitamin D in TB-infected individuals and has decades of study management experience. Dr. Ganmaa has conducted several studies in Mongolia, including a six-armed randomized trial of 744 participants, and 420 premenopausal women simultaneously in 2009. Dr. Spiegelman has many years of experience providing biostatistical expertise to international projects. As an ensemble, the investigators have experience in study design, monitoring, trouble-shooting and problem-solving that is required to run this trial.

7.5. Briefly describe how the research facilities and equipment at the research site(s) support the protocol's aims, e.g., private rooms available for interviews, etc.

The Mongolia project office will be based at the MNUMS (former HSUM), which has a long history of collecting and analyzing data and serves as a local study base for several US-funded research projects more than decade.

7.6. Are there provisions for medical and/or psychological support resources (e.g., in the event of incidental findings, research-related stress)?

☐**No** ☒**Yes: If yes; describe the provisions and their availability**

Children who choose to participate in the research study, and are found to have rickets and/or LTBI, will be referred for medical treatment. Additionally, full-time study doctors will be available to attend to the health and/or psychological needs of participating children, and the treatment of opportunistic infections.

8. Vulnerable Populations

8.1. Are there any potentially vulnerable populations (e.g., children, pregnant women, human fetuses, neonates, prisoners, elderly, economically disadvantaged, employees or students of the investigator or sponsor, undocumented, terminally ill, cognitively impaired or mentally ill, etc.)?

☐**No: If no, skip to 9.1**

☒**Yes: If yes; identify all vulnerable populations**

It is necessary to involve children for the trial study with primary endpoint of latent TB infection. LTBI is usually acquired in childhood following inhalation of TB bacteria from a person with infectious active TB. By the time they reach adulthood, approximately 60% of people in Mongolia will have acquired LTBI. In 10-20% of these people, LTBI will reactivate to cause active TB disease. Preventing people, especially children, from acquiring LTBI could therefore dramatically reduce the number of people who go on to develop active TB.

8.2. Describe safeguards to protect their rights and welfare

A letter with detailed information will be sent home to parents explaining the study and inviting parents and children to come to twenty-five informational meetings about the study (thus keeping each meeting to 40 parent/child pairs). Literacy rates are quite high in Mongolia (95% of adults can read) meaning that this sort of written communication is likely to be read and understood.

At the completion of the meeting and question and answer period parents and children will be invited into an adjacent private room where one of the study teams (Dr. Ganmaa, Project Coordinator or the research fellows) will ask if they have questions, and administer informed consent and assent forms for those who wish to proceed, with the school doctor as witness. It will be made clear that there would be no adverse ramifications if they chose not to participate. Families who wish for more time to consider participating will be contacted at a later time.

The research fellow will pose specific statements to the child, such as:
“ I agree that it is okay with me to do these things: (list study procedures).”
“ I understand that I am agreeing to do the things described. Also, I know that it is my choice to be in the study and I know I can decide not to be in the study anytime I want to.”
There will be repeated assurances throughout the study that any child may drop out at any time for any reason.

9. Consent Process

9.1. Will consent to participate be obtained?

☐**No: If no, skip to 9.4**

☒**Yes: If yes; describe the setting, role of individuals involved, timeframe(s), and steps to minimize coercion/undue influence during the consent process (at the time of initial consent and throughout the study)**

Informed consent will be obtained from each subject only by our full-time research fellows and will occur in 2 stages. First, at the initial encounter with a potentially eligible participant, she/he will be given an overview of the rationale and methods of the study. Research staff will clearly convey to each subject that participation is voluntary and that withdrawal from the study after consent will be granted without penalty. In addition to the study investigators, a TB Surveillance Center staff member will be designated as someone the participants may go to with any concerns regarding the study, or if they would like to terminate their participation in the trial.

The consent process will be conducted in the local language (Mongolian). Consent from each participant will be obtained in writing by research fellows who will then document that all necessary steps have been completed, regardless of whether the potential participant raised questions or concerns or if she/he granted consent.

Sufficient time will be provided to subjects to considering participation and consent; opportunities to consider the risks/benefits of participation will also be provided. To ensure the subjects' privacy and confidentiality during the consent process, a private room at participating schools will be available for use. Prior to the study, all research fellows will receive training in carrying out consent procedures and in working with human subjects. Research fellows participating in this study will preferably have previous experience in these areas. The study team will provide adequate time for questions, including those related to the practices employed successfully in the pilot study.

Are there any special populations?
☒No ☐Yes: If yes; describe the process to obtain consent, permission or assent

9.2. **Will consent of the participants be documented in writing?**
☒Yes ☐No: If no; describe the rationale for requesting a waiver or alteration of documentation of consent (and/or parental permission)

9.3. **Will participants be provided with a copy of their signed consent form or information sheet (when a consent form is not signed)?**
☒Yes ☐No: If no; explain any extenuating circumstances that make it impossible or inappropriate to meet this requirement, i.e., doing so may place participants at increased risk, if inadvertently disclosed

9.4. **Is a waiver or alteration of consent (and/or parental permission) being requested?**
☒No ☐Yes: If yes; describe the rationale for the request. If the alteration is because of deception or incomplete disclosure, explain whether and how participants will be debriefed (include any debriefing materials with the application)

10. Risks

10.1. **Are there any <u>reasonably foreseeable</u> risks, discomforts, and inconveniences to participants and/or groups/communities?**
☐No X☒Yes: If yes; indicate probability, magnitude, and duration of each (note that risks may be physical, psychological, social, legal, and/or economic)
Blood draw: Five ml blood samples will be taken from study participants to allow for testing for the presence of LTBI (QFT-G test), which requires a three ml blood sample, and testing for vitamin D status, which requires a 2 ml blood sample. The potential risks of blood drawing are hematoma and infection at the skin site, minimal pain of venous puncture, and uncommonly, fainting reactions. Discomfort resulting from phlebotomy will be minimized by prior application of local anesthetic cream to venipuncture sites. Other potential adverse

events: Another risk of participation in this study is the social-psychological risk resulting from inadvertent disclosure of medical history information.

Vitamin D Supplementation: Although vitamin D toxicity can occur, it is unlikely due to the doses being administered. The treatment is safe, and no case of vitamin D toxicity has ever been documented for cholecalciferol of 10,000 IU/day or higher. In addition, among those with high sun exposure, no case of vitamin D toxicity from sun exposure has ever been reported in the medical literature. For example, lifeguards can make the equivalent of 20,000 IU or higher of vitamin D in a day and achieve levels of 25(OH)D about twice as high as expected in this study, and yet they have not reported vitamin D toxicity. Cholecalciferol from oral intake is chemically identical to that formed from sun exposure. Further ensuring a low likelihood of toxicity is the likely relatively low baseline 25(OH)D in this population (35). In keeping with the observations above, a clinical study in schoolchildren comparing efficacy and safety of two different vitamin D regimens, equivalent to 200 IU/day vs. 2,000 IU/day administered for one year, found that the higher regimen was significantly more effective in correcting vitamin D deficiency. Those on the low dose regimen attained mean 25(OH)D levels of 19ng/ml (below the 30 ng/ml threshold of sufficiency) as compared with those on the higher dose who attained mean 25(OH)D levels of 36ng/ml (well above the 30 ngl/ml threshold of sufficiency) (34). Importantly, no child receiving either dosing regimen experienced hypercalcaemia or any other feature of vitamin D toxicity. Moreover, vitamin D repletion resulted in improvement in a range of musculoskeletal parameters (34). Because this study was of long duration (12 months, i.e. 24 half-lives of 25[OH]D), participants achieved steady state 25(OH)D concentrations for > 9 months. The fact that no toxicity was observed over this extended period provides strong reassurance that no toxicity is likely to be observed when this regimen is given for 3 years rather than 1 year.
Further evidence of the safety of administration of bolus doses of vitamin D comes from another study. The dose given to schoolchildren, with an average age of 8.6 years, in that study was more than twice what we have proposed (72), with 150,000 IU administered without induction of hypercalcaemia, hypervitaminosis D, or hypercalciuria.

Based on the principles above, doses equivalent to 2,000 IU daily will not elevate 25(OH)D concentrations into this range. Moreover, children receiving vitamin D supplementation are at particularly low risk of experiencing toxicity as they are experiencing rapid skeletal growth which is characterized by mild secondary hyperparathyroidism and a tendency for hypocalcaemia. Further evidence of the safety of the proposed dose comes from the latest United States Institute of Medicine guidelines on vitamin D intake (73), which state that the tolerable upper intake levels in children between 4 to 8 years and 9 to 13 years are 3000 IU/day (or 42,000 IU/bi-weekly) and 4000 IU/day (or 56,000 IU/bi-weekly), respectively. These doses are 1.5 to 2 times the proposed dose of 28,000 IU/ bi-weekly.

10.2. Identify whether any of the information collected, if it were to be disclosed outside of the research, could <u>reasonably</u> place the participant at risk of criminal or civil liability or be damaging to the participant's financial standing, employability or reputation.
N/A

10.3. Outline provisions in place to minimize risk
To assess safety among those who are eligible and enroll, participants will be informed of potential symptoms of hypercalcemia and asked to call the study coordinator if he or she experiences any such symptoms. Further, we will assess symptoms at the beginning of week of each month when the next capsule is provided as well as problems with adherence. Any symptoms noted will be reported to the study physician, who will make a determination if

any reported symptoms are possibly related to vitamin D toxicity. If toxicity due to hypercalciuria, hypervitaminosis D, or hypercalcemia is deemed a possibility, the participant will be asked to discontinue taking the pills and a blood sample for calcium will be taken. Phlebotomy will be performed using standard sterile technique. After the blood draw, traditional tea or soup will be served, as we did in the pilot study.

11. Benefits

11.1. Describe potential benefits of study participation (indicate if there is no direct benefit)

Benefits include the educational experience of participating in a research study. In our pilot study, teachers and school administrators were pleased with the exposure of the children to the scientific process. At the beginning of the pilot intervention, Dr. Ganmaa ran educational sessions with the pilot classroom to explain the research goals and how the children's participation contributed to these goals. We will do the same for the larger study. When we return to Mongolia to present results in Year 5 of the study, we will make presentations for families at each school, as we did for the pilot study.

Given the observational evidence that higher concentrations of vitamin D protect against several diseases including osteoporosis, osteomalacia, acute respiratory infections, hypertension, diabetes, and some cancers, intake of vitamin D supplementation in childhood may well prove to reduce future disease risk.

Subjects will receive growth monitoring and TB screening, which children would not otherwise be offered. Full-time study doctors will be available to attend to their health needs including free diagnosis and treatment of opportunistic infections.

11.2. Describe potential benefits of the research to the local community and/or society

For Mongolian society in particular, this study will: generate health data that Mongolia currently lacks, regarding the vitamin D status of Mongolian schoolchildren and the growth and developmental data for children in Ulaanbaatar; increase research infrastructure by launching the first large-scale international research collaboration, and by conducting the first longitudinal study of health in Mongolia. Individual scientists and students will gain first-hand experience in organizing and conducting studies, analyzing data, and interpreting results of this study. The scientists will participate in the DSMB to be run in Mongolia.

12. Reportable Events

12.1. Outline plans for communicating reportable events (e.g., adverse events, unanticipated problems involving risks to participants or others, breach of confidentiality)

If a child is sick, she/he will be evaluated by the local school doctor. The physician will report immediately to Dr. Ganmaa and Project Coordinator whether the illness/symptom reported is related to the trial. Dr. Ganmaa will report the details of the symptoms and the local doctor's opinion of the likelihood that the adverse event is related to participation in the trial ("likely association with participation", "unlikely association with participation", "unknown association with participation") to the ERB and to the HSPH OHRA. A case report of the adverse event will be generated and submitted to the HSPH OHRA and to the Ministry of Health ERB in Mongolia. We will communicate these events as quickly as possible. The only special circumstances are the time zone difference between Ulaanbaatar and Boston, which can slow communication. Serious adverse events that are unexpected and possibly related to the study including hypercalcemia and other events possibly related to study participation (such as an infection at the site of a research blood draw, or a breach of confidentiality) will be documented on the Reporting Form and reported within 24 hours by the Study Coordinator to Drs. Malchinkhuu and Ganmaa by email, or by phone if the internet is not working. Drs. Malchinkhuu and Ganmaa will confirm receipt of notice by email within 24 hours and will be reported to OHRA within 7 calendar days. Non-serious adverse events that are unexpected

and possibly related to the study will be reported to OHRA within 21 calendar days. All other adverse events will be reported at the time of continuing review. All unanticipated problems (non-medical occurrence) that involve risk to subjects or others will be reported to OHRA within 7 calendar days. In the case of other events possibly related to study participation, Dr. Ganmaa will consult with TSC (Drs. Willett, Martineau, Bloom, Giovannucci) to determine whether the event could possibly be related to study participation. Any event deemed to be the result of study participation will be reported to the DSMB and IRBs within 5 business days (within 7 calendar days). Serious adverse events that are NOT thought to be study related will be documented on the Reporting Form, which will be sent to Drs. Ganmaa and Malchinkhuu weekly, for them to verify that the event is not related to study participation. These events include any hospitalization, death; other serious illness. These events will be summarized for the review of the DSMB monthly. The DSMB will have access to the Vitamin D dose to which each subject has been randomized. Moderate adverse events that are NOT thought to be study related will be summarized for the review of the DSMB at their annual meeting. The DSMB will have access to the Vitamin D dose to which each subject has been randomized. The DSMB reports will be given to IRBs.

13. Research Related Injuries (this section must be completed for any greater than minimal risk research)

13.1 Are there provisions for medical care and compensation for research-related injuries?

☐**No** ☒**Yes: If yes; outline these provisions (Please note that although Harvard's policy is not to provide compensation for physical injuries that result from study participation, medical treatment should be available including first aid, emergency treatment and follow-up care as needed. If the research plan deviates from this policy, provide appropriate justification.)**

All individuals in Mongolia are covered by universal health insurance provided by the government. Although complications from a blood draw are highly unlikely, any major medical expenses incurred would be covered by the government-provided health insurance. The study will set aside up a small fund to treat minor symptoms related to the intervention as we did in our previous study.

14. Participant Privacy

14.1. Describe provisions to protect participants' privacy (their desire to control access of others to themselves, e.g., the use of a private interview room) and to minimize any sense of intrusiveness that may be caused by study questions or procedures

Confidentiality will be maintained by numerically coding data, by disguising identifying information, and by keeping all data in locked file drawers. All information obtained from subjects will be accessible only to research staff. All staff will be trained in confidentiality procedures, and routine refreshers provided.

15. Data Confidentiality

15.1. Will the information that is obtained be recorded in such a manner that participants can be identified, directly or through identifiers linked to the participants?

☐**No: If no, skip to <u>16.1</u>**

☒**Yes: If yes; either state that participants will be told that their data will be public or describe provisions to maintain the confidentiality of identifiable data, e.g., use of password protections (please refer to the Harvard Research Data Security Policy Protection Memo, at <u>http://www.security.harvard.edu/harvard-research-data-security-policy-protection-memo</u>, for additional information about required data security measures) [NOTE: Harvard Research Data Security Policy does not always apply if data are not being stored at Harvard facilities. Please consult the Data Security Policy for additional information.]**

Paper files kept in a locked cabinet in a locked office: the consent form with personally identifiable data (name, phone number, address, date of birth, and alternate contacts) in case we cannot reach participants; paper questionnaires, adverse event forms and any hard copies of laboratory results, identified only by study ID.

Electronic files will be kept on a password-protected computer.

A separate electronic file will be maintained on a password-protected computer that links patient name, date of birth, study ID, and assigned treatment arm at the HSUM office.

15.2. Describe i) whether data will be transmitted, and if so how; ii) how long it will be stored; and iii) plans for the data at the end of the storage period (how will it be destroyed, or will it be returned to data provider)

Data will be stored with identifiers until three years after the last follow-up visit in case in Mongolian or other researchers are interested in further follow up. After this time, the link between identifiers will be destroyed.

We will keep de-identified data at least 7 years after study closure to permit ample time for qualified and IRB-approved researchers and students to make full use of the dataset.

Data will be transmitted without patient identifiers as follows: by telephone or Skype communication, primarily in the case of a serious adverse event and by email between Ulaanbaatar, UK (co-PI Dr. Martineau's base) and Harvard.

15.3. Indicate how research team members and/or other collaborators are permitted access to information about study participants

Study doctors will have information regarding participants as part of routine care. The Study Coordinator will also have information access, which is necessary to complete adverse event forms. Harvard-based investigators, and DSMB members will not have access to identifiable information about study participants.

16. Costs and Payments

16.1. Identify any costs that participants may incur during the study, including transportation costs, childcare, or other out-of-pocket expenses

No cost and payment will incur to the participants during the study.

16.2. Is there any payment or reimbursement that participants may receive during the study?

☐No ☒Yes: If yes; specify the amount, method and timing of disbursement. (Please refer to Harvard University Financial Policy on Human Subject Payments at http://vpr.harvard.edu/sites/vpr.harvard.edu/files/news/Human%20Subject%20Payments%20Policy%20Final_0.pdf)

Vitamins will be provided at no cost to the participants.

17. Multi-site Study Management

17.1. Is this a multi-site study?

☒No ☐Yes: If yes; describe plans for communication among sites regarding adverse events, interim results, protocol modifications, monitoring of data, etc.

18. Investigational Drug/Biologic/Device

18.1. Does this study involve an Investigational Drug/Biologic/Device?

☒No: If no; skip to 19.1

☐Yes: If yes; identify and describe the drug/biologic/device (e.g., marketing status: Is there an IND/IDE, classification of a device as significant vs. non-significant risk)

18.2. Describe its administration or use

18.3. Compare the research drug/biologic/device to the local standard of care

18.4. Describe plans for receiving, storage, dispensing and return (to ensure that they will be used only for participants and only by authorized investigators)

18.5. If proven beneficial, describe anticipated availability and cost to participants post-study; plans (if applicable) to make available

19. HIPAA Privacy Protections

19.1. Are HIPAA privacy protections required? Please note that only Harvard University Health Services and Harvard School of Dental Medicine are covered entities at Harvard. Harvard is otherwise not a HIPAA covered entity. If, however, data is derived from a Covered Entity (e.g. a hospital or community health center), mark 'yes' and address the items below.

☒**No: If no; skip to** <u>**20.1**</u>

☐**Yes: If yes; include at least one of the following:**

Describe plans for obtaining authorization to access protected health information

Provide the rationale for a waiver of authorization or limited waiver of authorization request

20. Data and Specimen Banking

20.1. Does the study include Data and Specimen Banking?

☐**No: If no; skip to** <u>**21.1**</u>

☒**Yes: If yes; identify what will be collected and stored, and what information will be associated with the specimens**

Subsamples of whole blood for subsequent DNA extraction (subject to obtaining necessary funds) will be stored to characterize genetic variants that may be determinants of susceptibility to acquisition of LTBI. We would also like to store supernatants from QFT-G tests specifically for Mtb antigen-stimulated blood in subset of participants. Analysis of cytokine concentrations in these samples will have potential to answer many important questions, including: a) what are the effects of in vivo vitamin D supplementation on ex vivo responses to Mtb antigens, and b) is there an immunological phenotype that associates with resistance to acquisition of LTBI in the future

Describe where and how long the data/specimens will be stored and whether participants' permission will be obtained to use the data/specimens in other future research projects

The specimens will be stored for three more years after the completion the data collection and participants' permission will be obtained to use the data/specimens in other future research projects at the University of Health Sciences in Mongolia. At baseline, we will seek a tired permission from participants to test 25(OH)D levels, a set of other biomarkers that we know would be of interest if we could secure funding and an open-ended question regarding other analytes). Please see consent form.

20.2. Identify who may access data/specimens and how

Only members of the research team and the ethical review boards in Mongolia and the United States responsible for monitoring study safety have access to data. The participants will be identifiable only by study ID. We will not divulge data to employers or insurance companies.

Confidentiality will be maintained by numerically coding data, by disguising identifying information, and by keeping all data in locked file drawers. All information obtained from subjects will be accessible only to research staff. All staff will be trained in confidentiality procedures, and routine refreshers provided.

20.3. Will specimens and/or data be sent <u>to</u> research collaborators outside of Harvard?
☐No ☒Yes: If yes, describe the plan
The data will be sent to Dr. Adrian Martineau, co-PI for the proposal for quality assurance and regular checkups. All data will be stripped of identifiable information and coded by IDs only.

20.5 Will specimens and/or data be received <u>from</u> collaborators outside of Harvard?
☐No ☒Yes: If yes, describe the plan
We may send sub-samples of frozen samples from Mongolia to US. In this case, we will split all samples into several aliquots for frozen storage and this way we will insure that the safety of the sample shipment. One set of samples will be kept in the electrical -70°C freezer at the National Health Sciences University of Mongolia, and the other set of frozen samples from the study will be shipped to Prof. Sack's lab at HSPH.

21. Sharing Study Results

21.1. Is there a plan to share study results with individual participants?
☐No ☒Yes: If yes; describe the plan
We will communicate serum vitamin D results, results assessed by QTF-G to the participants and their parents. The parents and participants will have the option to not receive the results.

21.2. Is there a plan to disseminate aggregate results to the community where the research is conducted?
☐No ☒Yes: If yes, describe the plan
After data analysis, we will hold a last community meeting at the schools to explain the results to the community. Study personnel and study participants will be explicitly invited, and we will post notices in the community for other interested people to join. We will also seek to report the study results in national media.

22. Regulatory Compliance

22.1. Describe plan for monitoring regulatory compliance, in order to ensure proper record keeping and retention of required regulatory documents
Data will be stored with identifiers until 3 years after the last follow-up visit, at which point the link between identifiers will be destroyed. We will keep de-identified data at least 7 years after study closure.

2. Final Version of the Protocol

GENERAL INFORMATION	
**Protocol # (if assigned):** 14-0513	
**Version Date:** 04/25/19	**Version number:** v17.0
**Principal Investigator (PI): Davaasambuu Ganmaa**	
**Faculty Advisor (**if PI is a student**):**	
**Protocol Title: Vitamin D in TB Prevention in School Age Children**	

1. **Specific Aims**

Primary aim. To determine whether a weekly oral dose of 14000 IU vitamin D3 (equivalent to 2000 IU daily) reduces the risk of acquiring latent tuberculosis infection (LTBI) in children.

Secondary Aims .

1. To determine whether this regimen of vitamin D supplementation influences the following secondary efficacy outcomes:

a) in all participants:
• incidence of active TB disease
• incidence of self-reported acute respiratory infection (upper, lower and both combined)
• incidence of acute respiratory infection requiring hospitalization
• incidence of acute respiratory infections requiring antibiotic treatment
• number of days off school (total number and number due to acute respiratory infection)
• incidence of acute asthma exacerbation requiring hospitalization
• incidence of new asthma, allergic rhinitis and atopic dermatitis arising since baseline, based on ISAAC questionnaire data
• control of asthma, allergic rhinitis and atopic dermatitis identified at baseline, based on ISAAC questionnaire data
• incidence of bone fracture
• anthropometric outcomes (z-scores for height-for-age, weight-for-age, weight-for-height, body mass index-for-age, and waist circumference and waist-to-height ratio)
• body composition: impedance, impedance%, fat mass fat %, and fat-free mass
• muscle strength: grip strength and long jump distance from standing
• serum 25-hydroxyvitamin D concentration

b) in a sub-set of participants:
• bone mineral density at the radius
• physical fitness (maximal oxygen consumption estimated from 20m shuttle run)
• attention-related behavior scores (Connors III)
• prevalence of dental caries
• circulating and antigen-stimulated concentrations of cytokines, chemokines and other inflammatory mediators
• exam performance
• self-reported pubertal development
• urinary metabolome profile
• spirometric lung volumes (FEV1 and FVC)
• gut microbiome profile

2. To determine whether any effects of vitamin D supplementation on the outcomes above are modified by baseline vitamin D status, calcium intake or genetic variation in the vitamin D pathway (secondary outcome, efficacy).
3. To confirm that the regimen of vitamin D supplementation described above is safe and well-tolerated (secondary outcomes, safety)
4. To determine whether vitamin D supplementation is cost-effective for the prevention of LTBI and active TB.

2. Background

Provide the scientific background and rationale for the study

Tuberculosis (TB) is posing a global emergency. Not only is TB expected to surpass HIV as the largest single cause of death from infection in the period 1990 – 2020 (1), but multidrug resistant TB is on the rise (2), and an astonishing one-third of the global population, including 10-15 million people in the United States, are afflicted with a latent TB infection (LTBI) from *Mycobacterium tuberculosis* (*M. tuberculosis*). Young children are at the greatest risk for progression, while adolescents are also at increased risk (3). The mainstay for reducing transmission is treatment of active disease (4). However, mathematical models indicate that the WHO's 2050 elimination target cannot be met by this strategy alone (5), because most active TB arises as a consequence of reactivation of LTBI. LTBI is most commonly acquired in childhood, and reactivation occurs most commonly in adulthood. Thus, even if active TB is effectively treated, additional measures to prevent acquisition of LTBI in children will need to be implemented if the 2050 elimination target is to be met.

Classical approaches to preventing TB have focused on vaccination. While the existing TB vaccine, BCG, protects young children from disseminated disease, it is not generally considered to have a role in preventing acquisition of LTBI (6). New vaccine candidates are in development, but the lead times are very long, and the results of a recent trial have been disappointing (7). Alternative, low-cost strategies are needed to prevent acquisition of LTBI, particularly in schoolchildren in high TB transmission settings like Mongolia. Another very common condition in these settings is vitamin D deficiency (8), which a meta-analysis found it to be associated with susceptibility to acquisition and reactivation of LTBI (9). Vitamin D metabolites have long been recognized to boost innate immunity to *M. tuberculosis* in vitro (10), and Phase 2 clinical trials conducted by our team and others show that vitamin D supplementation boosts TB contacts' immunity to *M. tuberculosis* infection (11) and reduces schoolchildren's risk of acquiring LTBI by 59% (12).

An estimated 2.3 billion people worldwide have LTBI (13) and 1 billion are estimated to be vitamin D deficient (14). The WHO's Stop TB strategy aims to reduce incidence to <1 per million per year by 2050 (15). Mongolia has one of the largest number of incident TB cases in the world (16): the registered incidence of TB in Mongolia has increased from 79 to 141 per 100,000 between 1990 and 2001, and WHO estimates incidence at 230 per 100,000 Both *in vitro* and animal studies provide evidence of a protective relationship between vitamin D and the risk of acquiring latent TB infection. The evidence underpinning the trial we propose is compelling. Twenty-five years of *in vitro studies* have yielded the consistent finding, first suggested by Rook (17) and Crowle (18), that vitamin D metabolites induce innate immunity to mycobacteria *in vitro* via pleiotropic mechanisms, including induction of reactive nitrogen intermediates, reactive oxygen intermediates, antimicrobial peptides and autophagy (10). An *in vitro* study with human lung macrophages found that interferon gamma (IFN-γ) enhanced the killing activity of macrophages against *M. tuberculosis* in a dose-dependent manner, and that 1,25-D3, the biologically active form of vitamin D, synergistically augmented the effect of IFN-γ (19). More recently, Liu et al. showed that 25(OH)D supports TLR ligand-induced expression of the antimicrobial peptide cathelicidin and restriction of growth of intracellular M. tuberculosis (20). TLR induction led to cathelicidin activity, except when either the vitamin D receptor or the vitamin D activating enzymes were inhibited. Vitamin D deficiency has also been associated *in vivo* with increased progression of TB among infected mice and in cattle infected with *M. bovis* (21-23).

Human studies indicate that vitamin D deficiency may increase TB susceptibility (24,25). A systematic review of case-control studies found consistent independent associations between vitamin D deficiency and susceptibility to TB (9). Serum 25(OH)D was found to be lower in untreated TB patients than in healthy controls in the UK (26) and to be associated with the disease severity in Indonesia (27). Gibney (28) reported an association between higher vitamin D status and negative tuberculin status in asymptomatic people. Arnedo-Pena (29) showed that sufficient serum 25(OH)D concentrations are inversely associated with skin test conversion in a cohort study of people recently exposed to an infectious case. Despite substantial declines in TB in the United States, blacks continued to have TB at rates eight times greater than whites (30). Blacks also have virtually half the levels of 25(OH)D compared to whites, presumably largely due to lower synthesis in skin with greater melanin content, which blocks UV-B penetration. In racially integrated nursing homes, infection measured by tuberculin skin test conversion occurred twice as often in black as in white individuals who were equally exposed to active TB (31). In accordance with this, macrophages from blacks demonstrate a relative permissiveness for intracellular growth of virulent mycobacteria (32). A systematic review of observational studies reports that the association between vitamin D deficiency and susceptibility to TB is strong and widely replicated, and concluded that trials of vitamin D supplementation for TB prevention should be conducted.

Our primary hypothesis is that vitamin D supplementation will reduce risk of acquisition of LTBI in schoolchildren living in Ulaanbaatar, Mongolia.
Our secondary hypotheses are:

a) that vitamin D supplementation will reduce risks of active TB disease, acute respiratory infection, asthma exacerbation, incidence and control of atopic diseases (asthma, allergic rhinitis and atopic dermatitis), prevent bone fractures, improve growth and exam performance, and modify age of onset of puberty, bone mineral density and circulating markers of bone formation and modelling, body composition, muscle strength, physical fitness, spirometric lung volumes, attention-related behaviors, risk of dental caries, antimicrobial immune function, urinary metabolome profile, gut microbiome profile and vitamin D status
b) that biological effects of vitamin D supplementation on diverse health outcomes will be modified according to baseline vitamin D status, and genetic variation in vitamin D metabolism, transport and signaling pathways;
c) that a weekly oral dose of 14,000 IU vitamin D_3 is safe and well-tolerated; and
d) that vitamin D supplementation is cost-effective for the prevention of LTBI.

Our objective is to conduct a large (n=8,850) Phase 3 double-blind randomized placebo-controlled clinical trial powered to test these hypotheses.

Describe the significance of the research, and how it will add to existing knowledge
Two Phase 2 randomized placebo-controlled clinical trials have reported that *in vivo* vitamin D supplementation enhances antimycobacterial immunity. The first, conducted in London by our collaborator Dr Martineau, found that bolus-dose vitamin D supplementation taken by healthy people recently exposed to infectious TB cases enhanced the ability of their whole blood to restrict mycobacterial growth *ex vivo* by 25% (11). The second, conducted by our team in schoolchildren living in Mongolia (12), showed that vitamin D supplementation resulted in a 59% reduction in risk of tuberculin skin test (TST) conversion. While this finding suggests that vitamin D supplementation does indeed enhance innate immune resistance to LTBI, its interpretation is complicated by the fact that repeated tuberculin skin testing may lead to 'false positive' skin test conversions in the absence of LTBI (the boosting phenomenon). A more sensitive and specific means of diagnosing LTBI than the TST is to use interferon-gamma release assays (IGRA), which are not subject to the boosting phenomenon (33).

We therefore consider this the appropriate time to conduct a definitive Phase 3 clinical trial to determine whether vitamin D supplementation can prevent IGRA-diagnosed LTBI in populations living in high TB-transmission settings where vitamin D deficiency is very common. This research is significant, as positive results would mean an inexpensive, non-toxic means of reducing the incidence of a major global killer. The impact of this work will be particularly significant for children who live in high-risk TB settings, given that diagnoses of TB in children are difficult and children are at much greater risk of developing dangerous forms of disseminated disease such as miliary, or CNS, TB. In addition to their public health impact, the results will also inform clinical decision-making at the individual level, as the data will provide clinicians with an evidence base to inform their management of vitamin D-deficient patients who are at risk of TB. Impact from our research work could help in the design of preventive agents that would benefit millions of individuals and limit the spread of *M. tuberculosis* worldwide.

3. Study Setting

a. **Identify the sites or locations where the research will be conducted.**

Mongolia provides an excellent setting in which to conduct a study of this nature; vitamin D levels are low, supplementation is rare, the prevalence of TB is high, HIV co-infection is extremely low, the health care system is organized, participants are well educated and have enrolled in our studies in high numbers.

There are many reasons why 25(OH)D levels are low in Mongolia. Ulaanbaatar is located at a latitude of 48°N where UVB exposure from October through April is weak and temperatures preclude skin exposure. Furthermore, Mongolians do not typically consume vitamin D rich foods, their food supply is not fortified with vitamin D, and few people use vitamin D supplements.

b. Describe the Principal Investigator's experience conducting research at study site(s) and familiarity with local culture

Dr. Ganmaa has conducted several studies in Mongolia, including a six-armed randomized trial of 744 participants, and 420 premenopausal women simultaneously in 2009. As a co-investigator in another randomized, placebo controlled trial, she showed that vitamin D in a dose of 1000IU resulted in a modest, but statistically significant reduction in winter-related eczema in Mongolian children. As PI, Dr. Ganmaa completed a randomized clinical feasibility trial among Mongolian school-age children to test effect of vitamin D supplementation against latent TB infection. Currently, she is running three active studies on vitamin D in Mongolia. As a native Mongolian, she is familiar with the culture and system and thus she was able to successfully administer the research projects.

c. Is the research conducted outside the United States?

☒Yes: If yes; describe site-specific regulations or customs affecting the research, local scientific and ethical review structure

The proposal will be reviewed by the Institutional Review Board (OHRA) at Harvard T.H. Chan School of Public Health (HSPH), the Ethical Review Board (ERB) of the Ministry of Health (MOH) in Mongolia, the Ethical Committee of Health Sciences University of Mongolia, former name of Mongolian National University of Medical Sciences (MNUMS), and the Institutional Review Board of National University of Mongolia (NUM), FWA00012628.

d. Are there any permissions that have been or will be obtained from cooperating institutions, community leaders, or individuals, including approval of an IRB or research ethics committee? ☒Yes: If yes; provide a list of the permissions (also include copies with the application, if available)

We will apply for the Mongolian Ministry of Health ERB and National University of Mongolia IRB. As with our previous protocols in Mongolia, the ERB prefer for us to first receive HSPH approval before submitting for their review.

4. Study Design

a. Describe the study design type

We propose to conduct a double-blind, parallel-group individually randomized placebo-controlled, phase III trial of vitamin D to reduce the risk of TB infection among school-aged children in Ulaanbaatar, the capital of Mongolia.

b. Indicate the study's duration - and the estimated date of study completion

The study duration is five years with intervention running for three years. The estimated date of study completion is December 2019.

c. Indicate the total number of participants (if applicable, distinguish between the number of participants who are expected to be screened and enrolled, and the number of enrolled participants needed)

Approximately 12,000 subjects will be screened. We will randomize 8,850 participants in this trial.

d. List inclusion criteria

In order to maximize generalizability and representativeness, we deliberately set inclusion criteria to be broad. Eligible subjects are boys and girls without TB infection, ages 6 to 13, who are residents of Ulaanbaatar, who have given informed consent to participate in the study, whose parents/legal guardians have given informed consent, and who assent to participate in the study. Those testing positive for LTBI on screening will be assessed for active TB and referred for treatment. Children with clinical signs of rickets will be assessed by a study doctor and treated with vitamin D and calcium supplements.

e. List exclusion criteria

As necessitated by the study design, the presence of latent tuberculosis infection (LTBI) at screening will result in exclusion from the study. Children positive for LTBI on screening will be assessed for active TB and referred for treatment. Due to the high prevalence of TB in Mongolia, it will be not stigmatizing for those children who are ineligible due to positive LTBI.

Children with rickets at screening will also be excluded as to avoid randomizing those children to the placebo group. Additionally, the followings will be excluded:

- Known HIV sero-positivity, primary hyperparathyroidism or sarcoidosis at screening
- Taking immunosuppressant or cytotoxic therapy, vitamin D supplement > 10 micrograms/day, or 1-alpha-hydroxylated vitamin D preparations
- Plans to move away from study area within 3 years of enrollment

f. **Describe study procedures**

<u>Screening procedures and baseline data:</u> Assenting children whose parents/legal guardians also give consent for them to participate will be screened for eligibility to participate by trial staff. Those fulfilling all eligibility criteria will be allotted a unique interim ID code, consisting of a 2-digit 'school code' from 01 up to 22 (unique to each school), followed by a 4-digit 'participant code' (unique to each participant at that school, assigned consecutively in order of enrolment), followed by a four letter code (specific to first two letters of first name followed by first two letters of last name). Each part of this interim ID code will be separated by a dash (e.g. 01-0001-GADA, 01-0002-BAGU, etc).

Children will be screened at baseline by study doctors for symptoms or clinical signs of rickets (history of leg pain on walking, presence of wrist or ankle enlargement and / or knock-knee, bow-leg or windswept deformities of the legs). Those without such signs and symptoms will then undergo a baseline assessment, anthropometry, grip strength, long jump, whole

body impedance, and interviewed by research fellows detailing demographic and clinical data, including the ISAAC questionnaires for asthma, eczema and allergic rhinitis.
Physical fitness will be determined using the 20 meter multistage shuttle test (34) in a n=614 sub-sample of participants. BMD will also be determined using quantitative ultrasound bone densitometry in a separate n=1,464 sub-sample of participants. Attention deficit disorder/attention deficit hyperactivity disorder (ADD/ADHD) will be assessed using the Connors III test in n=201 sub-sample of participants. Dental caries questionnaire and examination will be conducted using a World Health Organization (WHO) Oral health surveys: basic methods-5 will be carried in n=220 sub-sample of participants. Baseline blood samples will be taken for vitamin D status, which will be determined in stored samples for all participants at the end of the trial. Participants will be screened for presence of LTBI at baseline, using a validated commercial whole blood assay, the QuantiFERON-TB Gold in-tube assay (QFT-G) and whole blood samples will be stored in deep freezer. The MNUMS, MHI and National Center for Communicable Diseases (NCCD) lab will provide a comprehensive package incorporating these services. When the result of the QFT-G test is available, participants with a negative QFT-G result will be randomized as detailed below, and invited to attend a second study visit for administration of first dose of vitamin D/placebo. Those with a positive QFT-G result will not be randomized, but they will also be invited to attend a second study visit to undergo screening for active TB.

Randomization: Participants will be randomized to receive vitamin D or placebo in a 1:1 ratio; randomization will be stratified by school. Randomization is stratified by school because school of attendance may be a strong risk factor for LTBI acquisition and we want to ensure that treatment assignment is balanced within this risk factor to avoid confounding in treatment effect estimation due to random imbalances. If a TB outbreak occurs in a particular school, children attending it might be at increased risk of acquiring LTBI. Prior to the start of recruitment, a statistician who is independent of the trial will prepare one school randomization.

The school randomization list will comprise paired 2-letter randomization codes (one pair of randomization codes per school, e.g. School 1: AA/AB, School 2: AC/AD etc). One randomization code within each pair will be randomly assigned to the active arm of the trial, and the other will be assigned to the placebo arm, using a computer-generated random sequence. The resulting school randomization list will be sent to Investigational Drug Services (IDS) at Brigham and Women's Hospital (BWH); copies will be held by members of the Data and Safety Monitoring Board (DSMB), and by a statistician Dr. Khudyakov and Prof. Chadraa who is independent of the trial, but participants and trial staff will not have access to it. The statistician who is independent of the trial will access this list in the event that a code break is required.

Preparation and use of participant randomization lists:
Each of the participant randomization lists will initially comprise of 6-digit numeric codes corresponding to participants' unique interim ID codes, i.e. consisting of a 2-digit school identifier from 01 up to 22, followed by a 4-digit participant identifier, separated by a dash (e.g. 01-0001, 01-0002). These 6-digit codes will then be randomly assigned to one or other of the 2-letter randomization codes in use for the corresponding school in blocks of ten, using a computer-generated random sequence. The resulting participant randomization lists will be supplied to the trial team, who will use them to append 2-letter randomization codes onto the end of eligible, randomized participants' interim ID codes, separated by a dash, to create a final ID code for each (e.g. if the participant randomization list indicates that participant 01-0001 has been assigned to randomization code AA, participant 01-0001-GADA's interim ID code will become final ID code 01-0001-GADA-AA; similarly, if the participant randomization list indicates that 01-0002 has been assigned to code AB, participant 01-002-BAGU's interim ID code will become final ID code 01-0002-BAGU-AB). Participants who

are ineligible for randomization will have the letters XX appended onto the end of their interim ID code to generate their final ID code. The enrolment of randomized participants will be documented in an enrolment log.

Intervention: The intervention will run over three school years. Parents will be asked to give study capsules during the summer and school holidays, and compliance will be monitored by counting the number of empty bottles returned.

The content of the vitamin D and placebo will be calibrated in advance by Tishcon Inc. One capsule of 14000 IU D3 will be given weekly on schooldays. If participants have missed taking softgel capsules, the trial staff will administer the missed softgels as a single dose, which may comprise up to four softgels given at one time. A field worker will be hired, trained, and assigned to each school as a ‘vitamin D monitor.’ These monitors will receive vitamin D/placebo capsules from study staff, and distribute them accordingly to participating children in their school. The monitors will observe that the vitamin D/placebo is consumed and will record any noncompliance. The project coordinator and a research fellow will supervise and retrain the ‘vitamin D monitors’ at regular intervals to ensure safety and maintain quality control. Prior to vacations scheduled to last for longer than 3 weeks, they will also supply sufficient capsules to allow weekly administration over the holiday period by participants’ parents or legal guardians. Study medication provided to parents / legal guardians for administration during holidays will be stored in the participants’ households.

Study Visits (see Table 1): Both the treatment and follow-up periods will be three years from enrolment. Administration of all doses of study medication will be directly observed by the study teams to optimize compliance and recorded in participants’ CRF.

Table 1. Study visits

Visit number	1	2	3	4	5	6	7	8	9	10	11	12	13	14
Visit timing	-1	0	3	6	9	12	15	18	21	24	27	30	33	36
Informed consent, assent	X													
Check eligibility criteria	X													
Record baseline data	X													
QFT-G	X													X
25(OH)D	X													X
Storage of whole blood	X													
Measure height	X					X				X				X
Measure weight	X		X	X	X	X	X	X	X	X	X	X	X	X
Measure waist circumference	X					X				X				X
Measure whole-body impedance	X					X				X				X
Measure grip strength	X					X				X				X
Long jump test	X					X				X				X
Cognitive test (exam performance)[3, 4] standardized														X
ISAAC questionnaires	X													X
BMD (n=1,464 subset)	X					X				X				X
Fitness test (multistage shuttle test; n=614 subset)	X					X				X				X
ADD/ADHD test (n=201 subset)	X													X

Dental caries (n=220 subset)	X													X
Randomization		X												
First dose of vitamin D		X												
Tanner stage[3], n=1200 at end-study														X
Calcium intake questionnaire										X	X	X	X	X
Fracture questionnaire										X	X	X	X	X
Diet/PDQS, n=1200 at end-study														X
Random spot urine sample[3], n=1200 at end-study														X
Spirometry[3], n=1200at end-study														X
Fecal samples[3], n=285														X

1. Study medication administered weekly 2. SAE, potential AR and symptoms of active TB (fever or cough of over 2 weeks' duration) will also be monitored at weekly follow-up. Study medication to be administered by parents during summer vacations. 3. At year 3 follow up only. 4. Grades with standardized test only

Anthropometric measurements (height, weight, BMI, waist circumference), ISAAC questionnaires, whole-body impedance, muscle strength (long jump, grip strength), bone mineral density (BMD), physical fitness, BMI for age will be determined at annual follow-up using validated scales and the portable height measure (UNICEF supply catalogue S0141021); BMI will be calculated as weight (kg)/[height (m)]^2. The video questionnaire on asthma symptom will be collected at the 36th month's visit and will be conducted after the ISAAC paper questionnaires. ADD/ADHD test and dental caries will be conducted at the first and 36th months of follow-up. The random spot urine sample, fecal sample collection, diet and spirometry test will be conducted at the 36th month.

Follow-up Procedures:

At weekly visits, study medication will be administered, and questions will be asked to elicit details of serious adverse events, symptoms suggestive of hypercalcaemia (nausea, vomiting, polydipsia, polyuria) and symptoms suggestive of active TB (fever or cough lasting >2 weeks) and those with history of recent household contact with an infectious TB case. Where such symptoms are reported, participants will be referred to the study doctor and at NCCD clinic if appropriate. Weekly administration of vitamin D and placebo, recording of Serious Adverse Events (SAE) will be performed on an on-going basis throughout the duration of the trial

At quarterly visits, weight will be measured and those who have lost more than 5% of the highest weight recorded in the past three months will be referred to the study doctor and at NCCD clinic if appropriate. Reasonable measures will be taken, as per the situation in each school, to ensure privacy and confidentiality.

At 12-month and 24-month visits, a more detailed assessment will be performed. For all participants, this will comprise a history of known TB exposures, measurement of weight, height, grip strength, whole body impedance, long jump, and waist circumference. Bone mass density will be measured for n=1,464 subset of participants. Fitness test (maximal oxygen consumption estimated from 20m shuttle run) will be measured for n=614 subset of participants.

At the 36-month visit, assessments will be performed as at 12- and 24-month visits. Additionally, all participants will be asked to give a 5 ml blood sample for a second QFT-G test and serum storage. Participants' in grades with only standardized mathematics examination (grades 5th and 9th) result will also be recorded, and those aged ≥10 years will be asked to complete the Tanner pubertal self-rating questionnaire. Subset of participants will take part in random spot urine collection, lung

function test, diet and fecal sample collection. N=201 subset of participants will be asked to take ADD/ ADHD test and n=220 subset of participants will be assessed for dental caries. Spirometry is a test used to assess lung function by measuring the amount and speed of air that can be inhaled and exhaled. During spirometry test, Forced Expiratory Volume in One Second (FEV1) and Forced Vital Capacity (FVC) will be tested.

At the final visit, results of the 36-month QFT-G test will be fed back to participants and their parents / legal guardians. Children found to have a positive QFT-G result at 36 months will undergo a clinical assessment by a study team member to screen for possible active TB. This clinical assessment will comprise a history (asking for symptoms of active TB) and a physical examination for signs of active TB, if indicated. Children with symptoms and/or signs suggestive of active TB will be referred to their local government TB clinic for further investigation and management..

Follow-up of children who develop chronic illness during the study

Children who develop a chronic illness during the course of the study will remain in follow-up and be included in the intention-to-treat analysis. They will continue to take study medication unless there is a medical contra-indication to doing so, such as being prescribed a prohibited concomitant therapy (namely supplemental vitamin D at a dose of >400 IU / day or equivalent, 1-alpha-hydroxylated vitamin D preparations such as alfalcalcidol or calcitriol, benzothiadiazine derivatives and cardiac glycosides).

End of Study Definition: The end of the study will be defined as the date 3 months after the day of the final visit of the final participant.

Procedures for unblinding

Unblinding will be conducted in accordance with the trial unblinding SOP, which will be available at site and with the person responsible for unblinding. A statistician who is independent of the trial will hold a copy of the School Randomization list. This statistician may unblind a participant's allocation in two scenarios:

1. During the course of the trial, in the following circumstances in case of a suspected severe adverse event (SAE) where knowledge of participant allocation may influence clinical management of a study participant

2. At the end of the trial, to allow data analysis.

In the event of an emergency code-break, measures will be taken to ensure that trial staff remain blinded to participant's allocation. On receipt of the treatment allocation details the PI or treating health care professional will continue to deal with the participant's medical emergency as appropriate.

g. **Does the study involve the collection of data/specimens (including the use of existing data/specimens)?**

☐**No** ☒**Yes: If yes; indicate how, when, where and from whom specimens or data will be obtained**

Blood samples at baseline and at the end of the three-year follow-up will be taken in the morning at the school clinic by experienced phlebotomists to assess 25(OH)-vitamin D levels. Serum 25(OH)D concentration will be determined by Biomeriuex/Vidas in batches at the end of the trial in stored serum from all participants. QFT-G test will be performed at baseline and at 3 years post-randomization.

h. **Is there a data and safety-monitoring plan (required for greater than minimal risk studies)?**

☐**No** ☒**Yes: If yes; describe the plan**

A data and safety monitoring board (DSMB) will be formed and will review interim safety and efficacy analyses at 12-month intervals. The DSMB will include independent U.S. and Mongolian scientists, statisticians, and clinicians with relevant experience. The DSMB will have the authority to stop any intervention arm if they determine that it is having an adverse impact on participant health. Trained data entry personnel will collate and clean the data

detailing all known serious adverse events to date in trial participants, and the study statistician, The study statistician will produce reports in accordance with the requests of the DSMB. Each meeting will include an administrative review to assess accrual, retention, and the progress of the study. In addition, there will be a blinded review of safety. A single efficacy analysis will be conducted on completion of the trial; no interim efficacy analyses will be conducted as data relating to the primary outcome (i.e. QFT-G test results) will not be available until the end of the study. Fatal and life-threatening significant adverse events (SAE) will be defined by the DSMB in consultation with the Trial Steering Committee (TSC). The DSMB will decide whether or not to unblind the study, and whether or not to consult with the sponsor and IRBs to decide whether the trial should be stopped, taking into account the severity of the SAE, biological plausibility, consistency with other study data, and the strength of the association.

DSMB Schedule: We propose that the DSMB meetings by conference call to decide the "stopping rule" protocol. A detailed Data and Safety Monitoring Plan (DSMP) will be submitted to the ERB and the Harvard Human Subjects Committee for approval prior to the recruitment of participants. The study biostatistician and PI will prepare reports for the DSMB, including data on participant recruitment, retention, compliance, and adverse events. The DSMB will meet at least annually during the course of the study. We propose the following members of the DSMB:

- Professor Michael Holick (expert on vitamin D, Boston University).
- Professor Robert Horsburgh (Epidemiology, Boston University School of Public Health) with extensive experience in TB for clinical trials
- Professor. Sarah Fortune (Immunology and Infectious Diseases, Harvard School of Public Health, TB expert the State Research Center on Maternal and Child Health, Mongolia).
- Professor Perenlei Enkhbaatar (University of Texas Medical Branch, Galveston, TX) has 12 years of clinical and teaching experience in lung disease both in Mongolia and the US.
- Dr. Erdenebaatar Chadraa (Assistant Professor of Mathematics, Minnesota State University).
- Dr. Paige L. Williams, biostatistician from Harvard T.H. Chan School of Public Health

In addition to the above measures, we will also convene a Scientific Advisory Board to advise the study. We will seek their input in evaluating risks and benefits, as well as the most effective ways to communicate risks and benefits (and eventually, study findings) to individuals and institutions participating in the study and to the public at large.

i. Are there any anticipated circumstances under which participants will be withdrawn from the research without their consent?
☒No ☐Yes: If yes; describe the circumstances as well any associated procedures to ensure orderly termination

Criteria for Premature Withdrawal
Participants will be withdrawn from the study if they withdraw assent to participate, if their parents / legal guardians withdraw consent for them to participate or if an investigator concludes that this course of action is in the participant's best interests. Participants will also be considered to have withdrawn if they consistently fail to attend scheduled study visits. If a participant withdraws, or is withdrawn, from the trial, the reason for withdrawal will be recorded in the case report form. When participants fail to attend scheduled study visits, members of the study team will make appropriate attempts to contact them
All participants who withdraw prematurely from the study will be invited for a final study visit at their earliest convenience. Final visit procedures will be carried out at this visit. No further follow-up will be arranged for withdrawn participants after this point. Data collected up to the time of withdrawal will be included in study analyses, unless otherwise requested by

a parent or legal guardian. The reason for withdrawal will be noted in the CRF. Any clinical samples taken prior to a participant's withdrawal will be used for study analyses

5. Data/Statistical Analyses Plan

a. Describe the plan for data analysis (including the statistical method if applicable)

To compare acquisition of LTBI in the intervention vs. control arms during the three-year follow-up, we will perform an intention-to-treat analysis. Differences in the proportion of children who acquire LTBI during the three-year period who were randomized to placebo will be compared to those randomized to the vitamin D regimen, using the Mantel-Haenszel risk ratio (24), stratified by school. The primary analysis will compare the proportion of children who are QuantiFERON-positive at the 0.35 IU/ml IFN-gamma threshold. Exploratory analyses will compare the proportion of children who are positive at the 4.0 IU/ml IFN-gamma threshold (denoting 'stable conversion') and mean / median antigen-stimulated IFN-gamma concentration analyzed as a continuous variable. No adjustment is needed for multiple comparisons or interim monitoring as the endpoint will be assessed only at the three-year end of study period. Other binary endpoints (e.g. proportions of participants experiencing hypercalcemia, hypercalciuria, hypervitaminosis D, treatment for active TB disease, confirmed/probable active TB as assessed by end-point committee, asthma exacerbation requiring hospitalization, pneumonia requiring hospitalization, new-onset asthma, eczema and allergic rhinitis, bone fracture) will also be analyzed using the Mantel-Haenszel risk ratio, stratified by school.

Continuous outcomes measured only once during follow-up, such as end-study 25-hydroxyvitamin D concentration, exam performance, and spirometric lung volumes, will be analyzed using linear regression, adjusted for school of attendance and baseline value, where available.

Continuous outcomes measured at more than one fixed timepoint (e.g. at 1, 2 and 3 years), such as BMI, whole-body impedance, grip strength, BMD and VO2 max (calculated from the number of laps completed in the shuttle run test using published formulae (34,35)) will be analyzed using linear regression, adjusted for school of attendance and for a random effect of individual. Outcomes with highly skewed distributions may be transformed prior to analysis. Adjusted treatment mean differences at different time-points will be presented with 95% confidence intervals. A P-value for treatment-time interaction will be used to evaluate evidence for an effect of treatment, and if evidence is found ($P<0.05$) then the P-values for the treatment effect at individual time-points will be reported.

Event rates (e.g. rate of upper respiratory infections, rate of school absence for acute respiratory infections) will be analyzed using negative binomial regression adjusted for school of attendance, accounting for the appropriate length of follow-up. The adjusted mean difference in rates between study arms will be presented with 95% confidence interval and P-value.

<u>Cost-effectiveness</u> of vitamin D supplementation for the prevention of LTBI / TB

We will conduct an economic evaluation to estimate the within-trial cost-effectiveness of using vitamin D to prevent LTBI. Rates of LTBI in the two study arms will be determined at 36 months post-randomization. Based on trial data, a decision analytic model will be developed to estimate the cost-effectiveness of vitamin D supplementation in the prevention of LTBI in schoolchildren compared to usual practice.

The model will be populated with probabilities of acquisition of LTBI. The cost of LTBI prevention will include costs of procuring, transporting, storing and administering vitamin D supplementation; costs of staff involved in vitamin D procurement and administration; and costs of producing information about vitamin D supplementation for parents/guardians. An incremental cost-effectiveness ratio will be estimated as cost per case of LTBI detected. One-way sensitivity analysis will be conducted to address the uncertainty associated with variation in costs and outcomes. Monte-Carlo simulations will be generated to access the overall uncertainty in model outputs. Any costs associated with serious adverse events will also be recorded.

Procedures to account for missing, unused and spurious data

The primary analysis of each outcome will include all participants with non-missing outcome data, and will adjust for the stratification factor (i.e. school of attendance) – a variable that will be non-missing for all study participants.

Pre-specified subgroup analysis

Heterogeneity of treatment effect will be examined among sub-groups defined by baseline vitamin D status, genotype and estimated calcium intake for both main trial outcomes and sub-study outcomes. Genotypic analyses will be conducted subject to obtaining necessary funds to conduct genetic studies. If such funds are obtained, DNA will be extracted from participants' stored whole blood, and typed for a panel of candidate single nucleotide polymorphisms (SNP) in genes influencing vitamin D metabolism, transport and signalling. The exact panel of SNP to be typed will be dependent on availability of funding; details of genetic analyses to be conducted will be provided in a substantial amendment to this protocol, to be made once funding for genetic studies has been secured.

Efficacy analyses may be repeated to include:

a) An interaction term between baseline vitamin D status and allocation to vitamin D vs. placebo

b) An interaction term between vitamin D pathway genotype and allocation to vitamin D vs. placebo

c) An interaction term between estimated calcium intake and allocation to vitamin D vs. placebo

The Benjamini-Hochberg procedure for multiple testing correction (36) will be applied to control the false discovery rate for genetic analyses at 20%.

Other pre-specified analyses

Subject to receipt of additional funding, we will conduct a case-control study to investigate environmental and genetic determinants of susceptibility to LTBI, utilizing data and DNA samples from QFT- G-positive participants ('cases') vs. QFT- G-negative participants ('controls') identified at screening.

We will also conduct a cohort study to investigate socio-demographic, clinical, genetic and transcriptional correlates of risk of acquisition of LTBI in study participants; this study will utilize genetic data obtained from baseline samples.
We will also compare acquisition of LTBI in participants during three-year follow-up according to serum 25(OH)D levels attained at the end of the study.

b. Is there a sample size/power calculation?

☐No ☒Yes: If yes; describe the calculation and the scientific rationale, and, if applicable, by site and key characteristics such as participant demographics

Styblo's Rule (36) predicts that for every 50 incident smear positive cases per year per 100,000 population, one would expect a 1% increase in Annual Risk of TB Infection (ARTI). WHO estimates the TB incidence in Mongolia at 230 per 100,000 per year. There is some disagreement about the current validity of Styblo's rule (37), particularly in areas of high HIV prevalence and low TB prevalence, but the Mongolian situation is opposite to that and more typical of TB epidemiology in the pre-HIV era: in Mongolia, the prevalence of TB is high, and HIV co-infection is extremely low. Using Styblo's Rule, and based on a TB incidence rate as estimated by WHO of 230 cases per 100,000 in Mongolia we estimate the annual ARTI rate in our study population will be 2% (44% of the 230 cases per 100,000 per year are smear positive) (36), which leads to an estimate that 5.9% of our subjects will become infected over 3 years of follow up. If, thinking conservatively, we expect vitamin D supplementation to reduce that predicted rate by 25%, as we assumed in our initial proposal; we get a predicted infection rate in the intervention arm of the study of 4.4% over 3 years.

Assuming 10% loss to follow-up (based on interviews with head teachers at participating schools and on our previous studies in Mongolia), and an additional 5% lost due to indeterminate results of the QFT-G at the end of the study, we originally estimated that we would need 4,010 subjects per arm, for a total of 8,020 (rounding up to the nearest ten subjects) to detect the hypothesized treatment effect with 80% power. Subsequently, we became concerned that rates of loss to follow-up might be higher than originally anticipated, and consequently the target sample size for the main trial was increased to 8,850.

For BMD, assuming standard deviation for BMD z-score of 1.1, a total of 952 participants (476 per arm) will need to be recruited and followed up to demonstrate a clinically significant difference of 0.2 z-scores between arms with alpha = 0.05 and power = 80%. Allowing for approximately 20% loss to follow-up, we originally estimated that a total of 1,200 participants (600 in each arm) would need to be recruited to the BMD sub-study. Subsequently, we became concerned that rates of loss to follow-up might be higher than originally anticipated, and consequently the target sample size for this sub-study was increased to 1464.

Analysis of circulating biomarkers of bone formation and modeling will use the same sample size as BMD analyses in order to maximize our ability to draw associations between BMD and serum biomarkers for each child, and thus better contextualize vitamin D's overall treatment effect on bone. End-study spirometry was also performed in participants in the BMD sub-study; the projected number of 1200 sub-study participants completing follow-up gives 86% power to detect a 70 ml difference in end-study FEV1, assuming a standard deviation of 0.4L.

For diet questionnaire, spirometry, urine and Tanner stage we will use will use the same participants and sample size as BMD

For the fitness studies, assuming standard deviation for VO2max of 6.5 ml/kg/min at 3-year follow-up, a total of 334 participants (167 per arm) will need to be recruited and followed up to demonstrate a clinically significant difference of 2 ml/kg/min between arms with alpha = 0.05 and power = 80%. Allowing for 20% loss to follow-up, we originally estimated that a total of 420 participants (210 per arm) would need to be recruited to the fitness sub-study. Subsequently, we became concerned that rates of loss to follow-up might be higher than originally anticipated, and consequently the target sample size for this sub-study was increased to 614.

6. Recruitment Methods

a. Does the study involve the recruitment of participants?

☐**No: If no, skip to <u>6.1</u>**

☒**Yes: If yes; indicate how, when, where, and by whom participants will be recruited**

There are 710 secondary schools in Mongolia, with a total of 557,346 children. Recruitment will take place over one year, in partnership with the Ministry of Education, Culture and Science of Mongolia (MECS) and the Government of Mongolia, which have responsibility over participating schools and have agreed to collaborate on this project (please refer to the enclosed letter). Participants will be recruited from up to twenty-two primary schools in Ulaanbaatar, selected on the basis of: lower socio-economic status (as these children are at potentially higher risk of acquiring LTBI and having baseline vitamin D deficiency); large size (as fewer schools will need to be visited in order to attain our target sample size, which reduces study costs); and proximity to the trial co-ordination center in Ulaanbaatar, reducing transport time and study costs. Based on an ARTI of 2% and results from feasibility trial (12), we estimate that 15% to 28% of 6-13 year olds will already have LTBI at screening, and will thus be ineligible for randomization; however, we will still be able to meet our target sample size.

Based on our experience of conducting clinical trials in children in Ulaanbaatar, and on interviews with school directors in participating schools, we estimate that no more than 10%

of otherwise eligible participants and/or parents will decline to take part in the study. Thus, in an average primary school with 700 pupils in primary schools, we anticipate that we will be able to randomize 500 pupils. Based on these assumptions, fifteen schools would need to be screened, and to ensure that our recruitment targets are reached, we have inflated this number to twenty-two schools.

b. Are there any materials that will be used to recruit participants, e.g., emails, posters, and scripts?

☐**No** ☒**Yes: If yes; provide a list of the materials (also include copies with the application)**

A letter will be sent home to parents explaining the study and inviting parents and children to come to informational meetings about the study. There are 3 to 8 meetings per day, thus keeping each meeting to 40 parent/child pairs. Literacy rates are quite high in Mongolia (95% of adults can read) meaning written communication is likely to be read and understood (Please also see enclosed script.)

7. Available Resources

a. Describe the feasibility of recruiting the required number of participants within the recruitment period

In Mongolia, a research team of 50 field workers, supervised by 4 research fellows, will be responsible for recruiting approximately 10,225 participants, and enrolling 8,850 of these. Additionally, they will perform weekly follow-up visits to the 14 or up to 22 participating schools, and pursue participants who do not attend these visits in order to minimize loss to follow-up. The field workers and research fellows will be supervised by a full-time trial coordinator and supported by a full-time project manager. Three study doctors will be responsible for assessing participants for development of active TB and fielding day-to-day medical queries from the study team. A team of five drivers will transport the study team to participating schools, and drive samples and participants requiring chest X-rays to the NCCD laboratory and clinical facilities in Ulaanbaatar.

b. Describe how the Principal Investigator will ensure that a sufficient amount of time will be devoted to conducting and completing the research

Dr. Ganmaa will have detailed oversight of day-to-day management of the trial in Mongolia and will coordinate applications for regulatory approval there. She will be assisted by a full-time trial coordinator, who will supervise a team of four research fellows and fifty fieldworkers responsible for recruitment and follow-up of study participants. A full-time project manager will also be employed to liaise with ethics and regulatory bodies, coordinate recruitment of the study team and handle administrative paperwork. On a daily basis, the project coordinator will communicate with the Boston office. Once weekly, Dr. Ganmaa, the project manager, coordinator, and local team will meet by Skype conference call. Dr. Ganmaa will oversee data collection/management; design, analyses, and travel to Mongolia; contribute to clinical interpretation of results; statistical analysis; and lead on write-up. She will also liaise with the study coordinator in Mongolia on a daily basis and the local staff and study doctors on a weekly basis, to perform study monitoring, and oversee laboratory services (NCCD lab) and data entry, to ensure that all study procedures comply with the sponsor-approved protocol, and will maintain oversight of study quality.

c. Are there research staff members, in addition to the Principal Investigator?

☐**No: If no, skip to** **6.5**

☒**Yes: If yes; outline training plans to ensure that research staff members are adequately informed about the protocol and study-related duties**

The Trial Steering Committee (TSC) with representation from investigators will be established with responsibility for overall management of the study. The committee will consist of Drs. Willett, Martineau, Bloom, Giovannucci, Spiegelman, Malchinkhuu, and

Ganmaa. They will lead on study design, provide expertise in nutrition trials, trial methodology, vitamin D and TB immunology and immunodiagnostics, and will contribute to clinical interpretation of results and write-up.The study statistician will prepare reports to the DSMB. Dr Ganmaa, Dr Martineau and the statistician will contribute to the statistical analysis plan, the statistical analysis, and write-up. Drs. Uyanga B and Batbayar O, former head of the NCCD TB Surveillance Division, will lead oversight and conduct of the trial in Mongolia. Dr James Seddon (Imperial College London) and Prof Ben Marais (University of Sydney) will form the end-point committee, which will review clinical and radiological data to classify cases of active TB into confirmed/probable/possible/unlikely disease; any disagreements between them will be resolved by discussion until a consensus is reached.

Collaboration: Our newly-formed partnership with the Ministry of Education, Culture and Science will be fostered by including a representative of the Ministry of Health, Trial Steering Committee (TSC), and by arranging 6-monthly meetings between Drs. Batbayar and Dr. Erkhembulgan, Deputy Director of Policy Implementation Department and Head of Education in Mongolian Ministry of Education, Culture and Science (MMECS). Professor Malchinkhuu has longstanding links with both the Ministry of Health as a chief expert pediatrician of the MMOH and Pediatric Specialty Committee of Mongolia. He will keep these organizations informed about progress of the trial as it proceeds. Both organizations have contributed to the design of the research.

d. **Describe the minimum qualifications for each research role (e.g., RN, social worker) their experience in conducting research, and their knowledge of local study sites and culture**

Drs. Martineau, Willett, Bloom, and Giovannucci have many years of experience in forming and following large cohorts and randomized clinical trials, both in Boston and internationally. Dr. Martineau is currently conducting several trials of vitamin D in TB-infected individuals and has decades of study management experience. Dr. Ganmaa has conducted several studies in Mongolia, including a six-armed randomized trial of 744 participants, and 420 premenopausal women simultaneously in 2009. Dr. Spiegelman has many years of experience providing biostatistical expertise to international projects. As an ensemble, the investigators have experience in study design, monitoring, trouble-shooting and problem-solving that is required to run this trial.

e. **Briefly describe how the research facilities and equipment at the research site(s) support the protocol's aims, e.g., private rooms available for interviews, etc.**

.

The Mongolia project office will be based at the MNUMS (former HSUM), which has a long history of collecting and analyzing data and serves as a local study base for several US-funded research projects for more than a decade.

The Mongolian Health Initiative (MHI), official subcontractor for Harvard T.H. Chan School of Public Health will be the primary coordinating center for the trial. Faculty and researchers at the MHI will contribute until the end of the project. The MHI will be the primary office for this study. The MHI will be responsible for the day-to-day data collection, including recruitment and follow up of study participants, interviews and measurements of study participants, abstraction of data from medical records, data entry, data cleaning, and collection, storage and shipment of biosamples, including coordination with local laboratories. The MHI faculty paid by this subcontract will also be responsible for preparing the materials for and making the presentation to the Ethical Review Boards of the Ministry of Health and the National University of Mongolia (Dr. Ganmaa will provide the protocol in English). MHI leadership will be responsible for financial management of this subcontract, including preparation of financial reports and invoices to the Boston office, which will work with the MHI leadership to establish financial management systems.

f. Are there provisions for medical and/or psychological support resources (e.g., in the event of incidental findings, research-related stress)?

☐**No** ☒**Yes: If yes; describe the provisions and their availability**

Children who choose to participate in the research study, and are found to have rickets and/or LTBI, will be referred for medical treatment. Additionally, full-time study doctors will be available to attend to the health and/or psychological needs of participating children, and the treatment of opportunistic infections.

8. Vulnerable Populations

a. Are there any potentially vulnerable populations (e.g., children, pregnant women, human fetuses, neonates, prisoners, elderly, economically disadvantaged, employees or students of the investigator or sponsor, undocumented, terminally ill, cognitively impaired or mentally ill, etc.)?

☐**No: If no, skip to <u>8.1</u>**

☒**Yes: If yes; identify all vulnerable populations**

It is necessary to involve children for the trial study with primary endpoint of latent TB infection. LTBI is usually acquired in childhood following inhalation of TB bacteria from a person with infectious active TB. By the time they reach adulthood, approximately 60% of people in Mongolia will have acquired LTBI. In 10-20% of these people, LTBI will reactivate to cause active TB disease. Preventing people, especially children, from acquiring LTBI could therefore dramatically reduce the number of people who go on to develop active TB.

b. Describe safeguards to protect their rights and welfare

A letter with detailed information will be sent home to parents explaining the study and inviting parents/legal guardians and children to come to informational meetings. There are about 3-8 informational meetings per day, thus keeping each meeting to 40 parent/child pairs. Literacy rates are quite high in Mongolia (95% of adults can read) meaning that this sort of written communication is likely to be read and understood.

At the completion of the meeting and question and answer period parents and children will be invited into an adjacent private room where one of the study teams (Dr. Ganmaa, Project Coordinator or the research fellows) will ask if they have questions, and administer informed consent and assent forms for those who wish to proceed, with the school doctor as witness. It will be made clear that there would be no adverse ramifications if they chose not to participate. Families who wish for more time to consider participating will be contacted at a later time.

The research fellow will pose specific statements to the child, such as:

"I agree that it is okay with me to do these things: (list study procedures)."

"I understand that I am agreeing to do the things described. Also, I know that it is my choice to be in the study and I know I can decide not to be in the study anytime I want to."

There will be repeated assurances throughout the study that any child may drop out at any time for any reason.

9. Consent Process

a. Will consent to participate be obtained?

☐**No: If no, skip to <u>8.4</u>**

☒**Yes: If yes; describe the setting, role of individuals involved, timeframe(s), and steps to minimize coercion/undue influence during the consent process (at the time of initial consent and throughout the study)**

Informed consent will be obtained from each subject only by our full-time research fellows and other trained study staff and will occur in 2 stages. First, at the initial encounter with a potentially eligible participant, she/he will be given an overview of the rationale and methods of the study. Research staff will clearly convey to each subject that participation is voluntary and that withdrawal from the study after consent will be granted without penalty. In addition to the study investigators, a TB Surveillance Center staff member will be designated as

someone the participants may go to with any concerns regarding the study, or if they would like to terminate their participation in the trial.

The consent process will be conducted in the local language (Mongolian). Consent from each participant will be obtained in writing by research fellows who will then document that all necessary steps have been completed, regardless of whether the potential participant raised questions or concerns or if she/he granted consent.

Sufficient time will be provided to subjects to considering participation and consent; opportunities to consider the risks/benefits of participation will also be provided. To ensure the subjects' privacy and confidentiality during the consent process, a private room at participating schools will be available for use. Prior to the study, all research fellows will receive training in carrying out consent procedures and in working with human subjects. Research fellows participating in this study will preferably have previous experience in these areas. The study team will provide adequate time for questions, including those related to the practices employed successfully in the pilot study. We will conduct consent addendum process including new study procedures such as spot urine samples, lung function test using spirometry, fecal samples for sub-set of participants and specifying language on the genetic testing and collected blood sample storage.

Are there any special populations?

☒No ☐Yes: If yes; describe the process to obtain consent, permission or assent

b. **Will consent of the participants be documented in writing?**
☒Yes ☐No: If no; describe the rationale for requesting a waiver or alteration of documentation of consent (and/or parental permission)

c. **Will participants be provided with a copy of their signed consent form or information sheet (when a consent form is not signed)?**
☒Yes ☐No: If no; explain any extenuating circumstances that make it impossible or inappropriate to meet this requirement, i.e., doing so may place participants at increased risk, if inadvertently disclosed

d. **Is a waiver or alteration of consent (and/or parental permission) being requested?**
☒No ☐Yes: If yes; describe the rationale for the request. If the alteration is because of deception or incomplete disclosure, explain whether and how participants will be debriefed (include any debriefing materials with the application)

10. Risks

a. **Are there any <u>reasonably foreseeable</u> risks, discomforts, and inconveniences to participants and/or groups/communities?**
☐No X☒Yes: If yes; indicate probability, magnitude, and duration of each (note that risks may be physical, psychological, social, legal, and/or economic)
Blood draw: Five ml blood samples will be taken from study participants to allow for testing for the presence of LTBI (QFT-G test), which requires a three ml blood sample, and testing for vitamin D status, which requires a 2 ml blood sample. The potential risks of blood drawing are hematoma and infection at the skin site, minimal pain of venous puncture, and uncommonly, fainting reactions. Other potential adverse events: possibility of gastrointestinal (GI) distress due to study medication intake and the social-psychological risk resulting from inadvertent disclosure of medical history information.

Vitamin D Supplementation: Although vitamin D toxicity can occur, it is unlikely due to the doses being administered. The treatment is safe, and no case of vitamin D toxicity has ever been documented for cholecalciferol of 10,000 IU/day or higher. In addition, among those with high sun exposure, no case of vitamin D toxicity from sun exposure has ever been reported in the medical literature. For example, lifeguards can make the equivalent of 20,000 IU or higher of vitamin D in a day and achieve levels of 25(OH)D about twice as high as

expected in this study, and yet they have not reported vitamin D toxicity. Cholecalciferol from oral intake is chemically identical to that formed from sun exposure. Further ensuring a low likelihood of toxicity is the likely relatively low baseline 25(OH)D in this population (38). In keeping with the observations above, a clinical study in schoolchildren comparing efficacy and safety of two different vitamin D regimens, equivalent to 200 IU/day vs. 2,000 IU/day administered for one year, found that the higher regimen was significantly more effective in correcting vitamin D deficiency. Those on the low dose regimen attained mean 25(OH)D levels of 19ng/ml (below the 30 ng/ml threshold of sufficiency) as compared with those on the higher dose who attained mean 25(OH)D levels of 36ng/ml (well above the 30 ngl/ml threshold of sufficiency) (39). Importantly, no child receiving either dosing regimen experienced hypercalcaemia or any other feature of vitamin D toxicity. Moreover, vitamin D repletion resulted in improvement in a range of musculoskeletal parameters (39). Because this study was of long duration (12 months, i.e. 24 half-lives of 25[OH]D), participants achieved steady state 25(OH)D concentrations for > 9 months. The fact that no toxicity was observed over this extended period provides strong reassurance that no toxicity is likely to be observed when this regimen is given for 3 years rather than 1 year.
Further evidence of the safety of administration of bolus doses of vitamin D comes from another study. The dose given to schoolchildren, with an average age of 8.6 years, in that study was more than twice what we have proposed (40), with 150,000 IU administered without induction of hypercalcaemia, hypervitaminosis D, or hypercalciuria.

Based on the principles above, doses equivalent to 2,000 IU daily will not elevate 25(OH)D concentrations into this range. Moreover, children receiving vitamin D supplementation are at particularly low risk of experiencing toxicity as they are experiencing rapid skeletal growth which is characterized by mild secondary hyperparathyroidism and a tendency for hypocalcaemia. Further evidence of the safety of the proposed dose comes from the latest United States Institute of Medicine guidelines on vitamin D intake (41), which state that the tolerable upper intake levels in children between 4 to 8 years and 9 to 13 years are 3000 IU/day (or 21,000 IU/ weekly) and 4000 IU/day (or 28,000 IU/ weekly), respectively. These doses are 1.5 to 2 times the proposed dose of 14,000 IU/ weekly.

There will be no additional risk from spirometry, urine test, fecal sample collection and additional questionnaires on incidence of bone fracture, diet, calcium intake and Tanner stage.

b. Identify whether any of the information collected, if it were to be disclosed outside of the research, could reasonably place the participant at risk of criminal or civil liability or be damaging to the participant's financial standing, employability or reputation.

N/A

c. Outline provisions in place to minimize risk

To assess safety among those who are eligible and enroll participants will be informed of potential symptoms of hypercalcaemia and asked to call the study coordinator if he or she experiences any such symptoms. Further, we will assess symptoms at each weekly visit when the next capsule is provided as well as problems with adherence. Any symptoms noted will be reported to the study physician, who will make a determination if any reported symptoms are possibly related to vitamin D toxicity. If toxicity due to hypercalciuria, hypervitaminosis D, or hypercalcaemia is deemed a possibility, the participant will be asked to discontinue taking the pills and a blood sample for calcium will be taken. Phlebotomy will be performed using standard sterile technique. After the blood draw, traditional tea or soup will be served, as we did in the pilot study.

11. Benefits

a. Describe potential benefits of study participation (indicate if there is no direct benefit)

Benefits include the educational experience of participating in a research study. In our pilot study, teachers and school administrators were pleased with the exposure of the children to

the scientific process. At the beginning of the pilot intervention, Dr. Ganmaa ran educational sessions with the pilot classroom to explain the research goals and how the children's participation contributed to these goals. We will do the same for the larger study. When we return to Mongolia to present results in Year 5 of the study, we will make presentations for families at each school, as we did for the pilot study.

Given the observational evidence that higher concentrations of vitamin D protect against several diseases including osteoporosis, osteomalacia, acute respiratory infections, hypertension, diabetes, and some cancers, intake of vitamin D supplementation in childhood may well prove to reduce future disease risk.

Subjects will receive growth monitoring and TB screening, which children would not otherwise be offered. Full-time study doctors will be available to attend to their health needs including free diagnosis and treatment of opportunistic infections.

b. Describe potential benefits of the research to the local community and/or society

For Mongolian society in particular, this study will: generate health data that Mongolia currently lacks, regarding the vitamin D status of Mongolian schoolchildren and the growth and developmental data for children in Ulaanbaatar; increase research infrastructure by launching the first large-scale international research collaboration, and by conducting the first longitudinal study of health in Mongolia. Individual scientists and students will gain first-hand experience in organizing and conducting studies, analyzing data, and interpreting results of this study. The scientists will participate in the DSMB to be run in Mongolia.

12. Reportable Events

a. Outline plans for communicating reportable events (e.g., adverse events, unanticipated problems involving risks to participants or others, breach of confidentiality)

If a child is sick, she/he will be evaluated by the local school doctor. The physician will report immediately to Dr. Unaganshagai Adiya, Dr. Maral Oyunsuren and Dr. Uyanga Buyanjargal whether the illness/symptom reported is related to the trial. Drs. Maral Oyunsuren and Uyanga will report the details of the symptoms and the local doctor's opinion of the likelihood that the adverse event is related to participation in the trial ("unrelated", "doubtful", "possible", "probable") to Project Coordinator, Manager, Dr. Erdenetuya and Dr. Ganmaa and they/she will report to the ERB and to the HSPH OHRA. A case report of the adverse event will be generated and submitted to the HSPH OHRA and to the Ministry of Health ERB in Mongolia. We will communicate these events as quickly as possible. The only special circumstances are the time zone difference between Ulaanbaatar and Boston, which can slow communication.

Serious adverse events that are unexpected and possibly related to the study including hypercalcemia and other events possibly related to study participation (such as an infection at the site of a research blood draw, or a breach of confidentiality) will be documented on the Reporting Form. We will test for hypercalcemia in any subject whose complaints of polyuria and/or polydypsia, are confirmed by an adult, or whose complaints of nausea and/or vomiting result in a visit to a health care provider.. Any subject who complains of polyuria and/or polydipsia will have a blood glucose (preferably fasting) measured in addition to serum calcium. A clinical record will be established that will record the date and nature of the complaints and all reposes to them, including whether or not blood tests are done, what the results are, and what actions are taken based on the results or complaints. Please refer to Reportable AE form.

We will refer any subject with high blood glucose to National Center for Maternal and Child Health of Mongolia (NCMCH) to diagnose and confirm juvenile diabetes. If juvenile diabetes is confirmed, we will continue to administer the study medication, if hypercalcaemia is confirmed, we will stop the study medication.

Dr. Maral or Dr. Uyanga will report the serious adverse events within 24 hours to Drs. Erdenetuya and Ganmaa by email, or by phone if the internet is not working. Drs. Erdenetuya and Ganmaa will confirm receipt of notice by email within 24 hours and will report to OHRA within 7 calendar days. All other adverse events will be reported at the time of continuing review. All unanticipated problems (non-medical occurrence) that involve risk to subjects or others will be reported to OHRA within 7 calendar days. In the case of other events possibly related to study participation, Dr. Ganmaa will consult with TSC (Drs. Willett, Martineau, Bloom, Giovannucci) to determine whether the event could possibly be related to study participation.

Any event deemed to be the result of study participation will be reported to the DSMB and IRBs within 5 business days (within 7 calendar days).

Serious adverse events that are NOT thought to be study related will be documented on the Reporting Form, which will be sent to Drs. Ganmaa, Malchinkhuu and Erdenetuya weekly, for them to verify that the event is not related to study participation. These events include any hospitalization, death and other serious illness. These events will be summarized for the review of the DSMB at their meeting. The DSMB will have access to the Vitamin D dose to which each subject has been randomized. Moderate adverse events that are NOT thought to be study related will be summarized for the review of the DSMB at their bi-annual meeting. The DSMB reports will be given to IRBs.

13. Research Related Injuries (this section must be completed for any greater than minimal risk research)

12.1 Are there provisions for medical care and compensation for research-related injuries?

☐**No** ☒**Yes: If yes; outline these provisions (Please note that although Harvard's policy is not to provide compensation for physical injuries that result from study participation, medical treatment should be available including first aid, emergency treatment and follow-up care as needed. If the research plan deviates from this policy, provide appropriate justification.)**

All individuals in Mongolia are covered by universal health insurance provided by the government. Although complications from a blood draw are highly unlikely, any major medical expenses incurred would be covered by the government-provided health insurance. The study will set aside up a small fund to treat minor symptoms related to the intervention as we did in our previous study.

14. Participant Privacy

a. Describe provisions to protect participants' privacy (their desire to control access of others to themselves, e.g., the use of a private interview room) and to minimize any sense of intrusiveness that may be caused by study questions or procedures

Confidentiality will be maintained by numerically coding data, by disguising identifying information, and by keeping all data in locked file drawers. All information obtained from subjects will be accessible only to research staff. All staff will be trained in confidentiality procedures, and routine refreshers provided.

15. Data Confidentiality

a. Will the information that is obtained be recorded in such a manner that participants can be identified, directly or through identifiers linked to the participants?

☐**No: If no, skip to 15.1**

☒**Yes: If yes; either state that participants will be told that their data will be public or describe provisions to maintain the confidentiality of identifiable data, e.g., use of password protections (please refer to the Harvard Research Data Security Policy Protection Memo, at http://www.security.harvard.edu/harvard-research-data-security-policy-protection-memo, for additional information about required data security**

measures) [NOTE: Harvard Research Data Security Policy does not always apply if data are not being stored at Harvard facilities. Please consult the Data Security Policy for additional information.]

Paper files kept in a locked cabinet in a locked office: the consent form with personally identifiable data (name, phone number, address, date of birth, and alternate contacts) in case we cannot reach participants; paper questionnaires, adverse event forms and any hard copies of laboratory results, identified only by study ID. Electronic files will be kept on a password-protected computer.

A separate electronic file will be maintained on a password-protected computer that links patient name, date of birth, study ID, and assigned treatment arm at the MHI office. Study data will be entered onto an on-line database via an electronic CRF using the REDCap web application. Staff will receive appropriate training to use the database and to complete the electronic CRF as necessary. In most instances, study staff will enter these data directly, either based on responses to questions that are asked of study participants or their parents / legal guardians, or from read-outs on study equipment (e.g. weighing scales). Laboratory data will be uploaded to the study database automatically to increased efficiency and avoid transcription errors.

The PI, co-investigators, data management team and study statistician will have access to study data during the trial. Study staff who require such access in order to conduct the trial will also be given such access as is necessary.

b. **Describe i) whether data will be transmitted, and if so how; ii) how long it will be stored; and iii) plans for the data at the end of the storage period (how will it be destroyed, or will it be returned to data provider)**

Data will be stored with identifiers until three years after the last follow-up visit in case in Mongolian or other researchers are interested in further follow up. After this time, the link between identifiers will be destroyed.

We will keep de-identified data at least 7 years after study closure to permit ample time for qualified and IRB-approved researchers and students to make full use of the dataset.

Data will be transmitted without patient identifiers as follows: by telephone or Skype communication, primarily in the case of a serious adverse event and by email between Ulaanbaatar, UK (co-PI Dr. Martineau's base) and Harvard.

c. **Indicate how research team members and/or other collaborators are permitted access to information about study participants**

Study doctors will have information regarding participants as part of routine care. The PI, Study Coordinator, local study team, study biostatistician will also have information access, which is necessary to complete adverse event forms.

16. Costs and Payments

a. **Identify any costs that participants may incur during the study, including transportation costs, childcare, or other out-of-pocket expenses**

No cost and payment will incur to the participants during the study.

b. **Is there any payment or reimbursement that participants may receive during the study?**

☐No ☒Yes: If yes; specify the amount, method and timing of disbursement. (Please refer to Harvard University Financial Policy on Human Subject Payments at http://vpr.harvard.edu/sites/vpr.harvard.edu/files/news/Human%20Subject%20Payments%20Policy%20Final_0.pdf)

Vitamins will be provided at no cost to the participants.

17. Multi-site Study Management

a. Is this a multi-site study?

☒No ☐Yes: If yes; describe plans for communication among sites regarding adverse events, interim results, protocol modifications, monitoring of data, etc.

18. Investigational Drug/Biologic/Device

a. Does this study involve an Investigational Drug/Biologic/Device?

☐☒No: If no; skip to **18.1**

Yes: If yes; identify and describe the drug/biologic/device (e.g., marketing status: Is there an IND/IDE, classification of a device as significant vs. non-significant risk)

b. Describe its administration or use

c. Compare the research drug/biologic/device to the local standard of care

d. Describe plans for receiving, storage, dispensing and return (to ensure that they will be used only for participants and only by authorized investigators)

Study medication (trial stock) will be manufactured by Tishcon Corp, USA, and imported into Mongolia. Mongolian Health Initiative (MHI), local NGO and HSPH subcontractor in Mongolia will store trial medication in a temperature-monitored environment, from which medication will be dispensed for administration to trial participants.

Active / placebo softgels will be administered over a 3-year period. During school term time, weekly administration will be supervised by the trial team. Immediately prior to any school holiday scheduled to last two weeks or less, additional doses of study medication will be administered to participants, amounting to one additional softgel capsule per anticipated week of absence from school, up to a maximum of four in total. For example, if participants are scheduled to have a vacation of two weeks' duration, they will receive a single dose of three softgels on the study visit immediately prior to this vacation. Administration of this dose of vitamin D_3 (42,000 IU) is safe: much larger bolus doses of vitamin D have been administered to much younger children without adverse effect (e.g. 100,000 IU bolus doses of vitamin D_3 were administered to infants without adverse effect in a large randomized controlled trial).(47)

Active / placebo softgels will be administered by the participants' home address in following condition:

1. If the child was absent at school and did not receive her/his study medications for summer or seasonal vacation
2. If the child has been absent at school because of illness or other reasons and missed 3 doses
3. If the child transferred to a new school
4. If the child lost the study medications during the vacation period
5. If any unforeseen circumstances such as school strike happen in the future,

For vacations and any unforeseen circumstances that are scheduled to last longer than three weeks (e.g. the December-January holiday), participants' parents / legal guardians will be supplied with sufficient softgels to enable them to administer study medication to their child on a weekly basis until s/he returns to school. Parents and legal guardians will be asked to store study medication at temperatures ≤ 25ºC, and medication will be provided in containers labelled with this instruction. During school holidays and any unanticipated conditions lasting longer than three weeks, study staff will provide parents / legal guardians with telephone or SMS reminders to administer study medication to participants in their care.Parents/ legal guardians of the students who transferred to different schools will be given ten to twelve study medications to administer their child's intake of the study medication on a weekly basis until the next delivery. The number of softgels that needs to be delivered will be based on the transferred students' distance and their legal guardians' possibility to come and receive it in person at child's school or at MHI

office. Study staff will provide parents/legal guardians with a proper guidance on administering study medication to their children through a telephone call or SMS in order to prevent from overdose. Thus, ask follow-up questions on a bi-weekly basis. Transferred students study medication administration and follow up procedures will be performed according to the relevant SOP.

Accountability/Receipt /Storage and Handling of active Investigational Medicinal Product (IMP) and placebo

Shipments of study medication from Tishcon Corp. to MHI, will be logged at points of departure and arrival. On arrival at the MHI, supplies of active IMP and placebo will be stored in the MHI until the scheduled date of administration of each dose. Administration of softgels to individual participants will be directly observed, and recorded in participants' CRF. Where participants have missed softgels, trial staff will administer these missed softgels in a single dose, which may comprise up to four softgels given at one time. Study medication provided to parents / legal guardians for administration during longer holidays will be stored in households.

Dispensing of active IMP and placebo

Study Medication Administration (SMA) staff at the MHI will dispense containers of softgels scheduled for administration to participants at that school on that day. Immediately prior to vacations scheduled to last for longer than 3 weeks, they will also supply sufficient capsules to allow weekly administration over the holiday period by participants' parents or legal guardians.

Stability of active IMP and placebo

Stability testing performed by Tishcon Corp indicates that active IMP and placebo are stable at temperatures ≤25ºC for 3 years.

Prior and Concomitant Therapies

Prohibited prior therapies are: Supplemental vitamin D at a dose >400 IU daily or equivalent in the previous month Any long-term medication other than asthma medication

Prohibited concomitant therapies are: Supplemental vitamin D at a dose of >400 IU / day or equivalent

1-alpha-hydroxylated vitamin D preparations (e.g. alfalcalcidol, calcitriol)

Benzothiadiazine derivatives

Cardiac glycosides

Dose modification/reduction/ delay

Administration of IMP may be delayed if a participant experiences symptoms of hypercalcaemia, pending results of a serum corrected calcium test. If a dose of IMP is missed or delayed, it can be administered together with the next scheduled dose of IMP. Up to four doses of IMP may be administered at one time if necessary, as outlined above.

Administration of IMP will be discontinued in the following circumstances:

1. If a participant is prematurely withdrawn from the study for any of the reasons stated in this protocol
2. If a participant develops hypercalcaemia (calcium concentration >2.65 mmol/L) confirmed on two consecutive blood samples
3. If a participant develops a condition which requires treatment with a prohibited concomitant therapy
4. If a participant develops a condition which, in the judgement of an investigator, adversely affects that participant's safety, compliance or ability to complete evaluations
5. If an investigator concludes that this course of action is in the participant's best interests.

Return/Recall or Destruction of IMP

Members of the study team will destroy expired or unused study medications according to local standard operating procedures.Arrangements for post-trial access to IMP and care

Participants who are found to be Quantiferon-positive on completion of the study will undergo a clinical assessment by a study team member to screen for possible active TB. This clinical assessment will comprise a history (asking for symptoms of active TB) and a physical examination for signs of active TB, if indicated. Children with symptoms and/or signs suggestive of active TB will be referred to the National Center for Communicable Disease, TB clinic for further investigation and management.

e. If proven beneficial, describe anticipated availability and cost to participants post-study; plans (if applicable) to make available

19. HIPAA Privacy Protections

a. Are HIPAA privacy protections required? Please note that only Harvard University Health Services and Harvard School of Dental Medicine are covered entities at Harvard. Harvard is otherwise not a HIPAA covered entity. If, however, data is derived from a Covered Entity (e.g. a hospital or community health center), mark 'yes' and address the items below.

☒**No: If no; skip to 19.1**

☐**Yes: If yes; include at least one of the following:**

Describe plans for obtaining authorization to access protected health information

Provide the rationale for a waiver of authorization or limited waiver of authorization request

20. Data and Specimen Banking

a. Does the study include Data and Specimen Banking?

☐**No: If no; skip to 20.1**

☒**Yes: If yes; identify what will be collected and stored, and what information will be associated with the specimens**

Subsamples of whole blood for subsequent DNA extraction (subject to obtaining necessary funds) will be stored to characterize genetic variants that may be determinants of susceptibility to acquisition of LTBI. We would also like to store supernatants from QFT-G tests specifically for Mtb antigen-stimulated blood in subset of participants. Analysis of cytokine concentrations in these samples will have potential to answer many important questions, including: a) what are the effects of in vivo vitamin D supplementation on ex vivo responses to Mtb antigens, and b) is there an immunological phenotype that associates with resistance to acquisition of LTBI in the future

Describe where and how long the data/specimens will be stored and whether participants' permission will be obtained to use the data/specimens in other future research projects

The specimens will be stored for three more years after the completion the data collection and participants' permission will be obtained to use the data/specimens in other future research projects at the MNUMS. At baseline, we will seek a tired permission from participants to test 25(OH)D levels, a set of other biomarkers that we know would be of interest if we could secure funding and an open-ended question regarding other analytes). Please see consent form.

b. Identify who may access data/specimens and how

Only members of the research team and the ethical review boards in Mongolia and the United States responsible for monitoring study safety have access to data. The participants will be identifiable only by study ID. We will not divulge data to employers or insurance companies. Confidentiality will be maintained by numerically coding data, by disguising identifying information, and by keeping all data in locked file drawers. All information obtained from

subjects will be accessible only to research staff. All staff will be trained in confidentiality procedures, and routine refreshers provided.

c. Will specimens and/or data be sent to research collaborators outside of Harvard?
☐No ☒Yes: If yes, describe the plan
The data will be sent to Dr. Adrian Martineau, co-PI for the proposal for quality assurance and regular checkups. All data will be stripped of identifiable information and coded by IDs only.

19.4. Will specimens and/or data be received from collaborators outside of Harvard?
☐No ☒Yes: If yes, describe the plan
We may send sub-samples of frozen samples from Mongolia to US. In this case, we will split all samples into several aliquots for frozen storage and this way we will insure that the safety of the sample shipment. One set of samples will be kept in the electrical -70°C freezer at the MNUMS, and the other set of frozen samples from the study will be kept in the deep freezer at MHI lab,.

21. Sharing Study Results
a. Is there a plan to share study results with individual participants?
☐No ☒Yes: If yes; describe the plan
We will communicate serum vitamin D results, results assessed by QFT-G to the participants and their parents/legal guardians. The parents/legal guardians and participants will have the option to not receive the results.

b. Is there a plan to disseminate aggregate results to the community where the research is conducted?
☐No ☒Yes: If yes, describe the plan
After data analysis, we will hold a last community meeting at the schools to explain the results to the community. Study personnel and study participants will be explicitly invited, and we will post notices in the community for other interested people to join. We will also seek to report the study results in national media.

22. Regulatory Compliance
a. Describe plan for monitoring regulatory compliance, in order to ensure proper record keeping and retention of required regulatory documents
Data will be stored with identifiers until 3 years after the last follow-up visit, at which point the link between identifiers will be destroyed. We will keep de-identified data at least 7 years after study closure.
The sites will be monitored by experienced Data Management Team (DMT) who will be assisted by the Project Coordinator (PC) and Project Manager (PM).

Regular monitoring visits to the schools and lab will be made to ensure that the trial is conducted and documented properly and that all aspects of the trial are followed, including:

- That the protocol is being followed,
- That facilities and staffing remain acceptable,
- That informed consent and assent has been conducted appropriately and correctly,
- That source documents are being correctly and adequately completed and data entered into the e-CRF database correctly,
- That the clinical supplies (including the test products) are accurately accounted for,
- That the test products have been transported to the schools according to requirements and are stored properly at the ,
- That the Investigator Site Files are up to date and maintained,

- That technical procedures such as participant Identification, phlebotomy technique and specific sample preparation techniques are performed according to relevant SOPs.

The first monitoring will occur within 3 months after the first participant randomized and again at months 6, 12, 24 and 36.

Audit and Inspection

Internal audits may be conducted by the SDM, and/or by an authorized external organization such as the MNUMS. The Sponsor retains the right to audit any trial, trial site or central facility. In addition, any part of the trial may be inspected by regulatory bodies and / or the funder where applicable.

References

1. Sherkat R. Mycobacterial infection in chronic granulomatous disease. Clinical & Experimental Allergy. 2004;34: 1806.
2. Pablos-Mendez A, Raviglione MC, Laszlo A, Binkin N, Rieder HL, Bustreo F, Cohn DL, Lambregts-van Weezenbeek CSB, Kim SJ, Chaulet P, Nunn P, for the World Health Organization-International Union Against Tuberculosis and Lung Disease Working Group on Anti-Tuberculosis Drug Resistance Surveillance. Global surveillance for antituberculosis-drug resistance, 1994-1997. N Engl J Med 1998;338:1641-1649.
3. Perez-Velez CM and Marais BJ. Tuberculosis in Children N Engl J Med 2012;367:348-61.
4. Raviglione MC & Uplekar MW. WHO's new Stop TB Strategy. Lancet 2006; 367, 952-955.
5. Dye C. & Williams BG. Eliminating human tuberculosis in the twenty-first century. Journal of the Royal Society, Interface / the Royal Society 2008; 5, 653-662.
6. WHO. BCG in immunization programmes. Weekly epidemiological record / Health Section of the Secretariat of the League of Nations 2001;76, 33-39.
7. Tameris MD, Hatherill M, Landry BS, Scriba TJ, Snowden MA, Lockhart S, Shea JE, McClain JB, Hussey GD, Hanekom WA, Mahomed H, McShane H. Safety and efficacy of MVA85A, a new tuberculosis vaccine, in infants previously vaccinated with BCG: a randomised, placebo-controlled phase 2b trial. Lancet 2013 Feb 1, EPub ahead of print.
8. UNICEF/Ministry of Health (1993) Child Nutrition Survey. Ulaanbaatar: UNICEF/Ministry of Health and Social Welfare.
9. Nnoaham KE. & Clarke A. Low serum vitamin D levels and tuberculosis: a systematic review and meta-analysis. Int J Epidemiol 2008; 37, 113-119.
10. Martineau AR. Old wine in new bottles: vitamin D in the treatment and prevention of tuberculosis. Proc Nutr Soc 2012; 71, 84-89.
11. Martineau AR, Wilkinson RJ, Wilkinson KA, Newton SM, Kampmann B, Hall BM, Packe GE, Davidson RN, Eldridge SM, Maunsell ZJ, Rainbow SJ, Berry JL, Griffiths CJ. A single dose of vitamin D enhances immunity to mycobacteria. Am J Respir Crit Care Med 2007; 176, 208-213.
12. Ganmaa D, Giovannucci E, Bloom BR, Fawzi W, Burr W, Batbaatar D, Sumberzul N, Holick MF, Willett WC. Vitamin D, tuberculin skin test conversion, and latent tuberculosis in Mongolian school-age children: a randomized, double-blind, placebo-controlled feasibility trial. Am J Clin Nutr 2012; 96:391-6.
13. Dye C, Scheele S, Dolin P, Pathania V. & Raviglione MC. Consensus statement. Global burden of tuberculosis: estimated incidence, prevalence, and mortality by country. WHO Global Surveillance and Monitoring Project. Jama 1999; 282, 677-686.
14. Holick MF & Chen TC. Vitamin D deficiency: a worldwide problem with health consequences. Am J Clin Nutr 2008; 87, 1080S-1086S.
15. WHO. The Stop TB strategy. (Geneva, 2006).
16. World Health Organization. Global Tuberculosis Control: Surveillance, Planning, Financing: WHO Report 2004. Geneva, Switzerland. http://www.who.int/tb/publications/global_report/en/
17. Rook GAW, Steele J, Fraher L, Barker S, Karmali R, O'Riordan J. Vitamin D3, gamma interferon, and control of proliferation of Mycobacterium tuberculosis by human monocytes. Immunology 1986; 57:159-163.
18. Crowle AJ, Ross EJ, May MH. Inhibition by 1,25(OH)2-vitamin D3 of the multiplication of virulent tubercle bacilli in cultured human macrophages. Infect.Immun. 1987; 55:2945-2950.

19. Bar-Shavit Z, Noff D, Edelstein S, Meyer M, Shibolet S, Goldman R. 1,25-duhydrohy-vitamin D3 and the regulation of macrophage function. Calcif Tissue Int. 1981; 33:673-76.
20. Liu PT, Stenger S, Li H, Wenzel L, Tan BH, Krutzik SR, Ochoa MT, Schauber J, Wu K, Meinken C, Kamen DL, Wagner M, Bals R, Steinmeyer A, Zugel U, Gallo RL, Eisenberg D, Hewison M, Hollis BW, Adams JS, Bloom BR, Modlin RL. Toll-like receptor triggering of a vitamin D-mediated human antimicrobial response. Science 2006; 311:1770-1773.
21. Waters WR, Palmer MV, Nonnecke BJ, Whipple DL, Horst RL. Mycobacterium bovis infection of vitamin D-deficient NOS2-/- mice. Microb Pathog. 2004;36(1):11-7.
22. Ametaj BN, Nonnecke BJ, Horst RL, and Beitz DC. Effects of retinoic acid and 1,25-dihydroxyvitamin D3 on IFN-γ secretion by mononuclear leukocytes from nulliparus and postparturient dairy cattle. Int. J. Nutr. Res. 2000; 70:92-101.
23. Waters WR, Nonnecke BJ, Rahner TE, Palmer MV, Whipple DL, and Horst RL. Modulation of Mycobacterium bovis-specific responses of bovine peripheral blood mononuclear cells by 1,25-dihydroxyvitamin D3. Clin. Diagn. Lab. Immunol. 2001; 8:1204-1212.
24. Davies PD. Tuberculosis and migration. The Mitchell Lecture 1994. J R Coll Phys Lond. 1995; 29:113-18.
25. Wilkinson RJ, Pasvol G. Tuberculosis, HIV, hormones and children. J R Coll Phys Lond. 1995; 29:86-88.
26. Davies PDO, Brown RC, Woodhead JS. Serum concentrations of vitamin D metabolites in untreated tuberculosis. Thorax 1985; 40: 187-90.
27. Davies PDO, Church HA, Bovornkitti S, Chatumilind A & Byrachandra S. Altered vitamin D homeostasis in tuberculosis. Inetrn Med (Thailand) 1988; 4:45-7
28. Gibney KB, MacGregor L, Leder K, Torresi J, Marshall C, Ebeling PR, Biggs BA. Vitamin D deficiency is associated with tuberculosis and latent tuberculosis infection in immigrants from sub-Saharan Africa. Clin Infect Dis 2008;46, 443-446.
29. Arnedo-Pena A, Juan-Cerdán JV, Romeu-Garcia A, Garcia-Ferrer D, Holguín-Gómez R, Iborra-Millet J, Herrero-Carot C, Piñana MJ, Bellido-Blasco J, Ferrero-Vega JA, Adsuara LS, Silvestre ES, Ferrer NM, Bartual VR.. Latent tuberculosis infection,tuberculin skin test conversion and vitamin D status in contacts of tuberculosis patients: a cross-sectional and case-control study. BMC infectious diseases 2011;11, 349.
30. CDC. Reported tuberculosis in the United States, 2002. Atlanta, Georgia: U.S. Department of Health and Human Services, CDC, 2003.
31. Stead WW, Senner JW, Reddick WT, and Lofgren JP. Racial differences in susceptibility to infection by Mycobacterium tuberculosis. N. Engl. J. Med. 1990; 322:422-427.
32. Crowle AJ and Elkins N. Relative permissiveness of macrophages from black and white people for virulent tubercle bacilli. Infect. Immun. 1990; 58:632-638.
33. Leyten EM, Prins C, Bossink AW, Thijsen S, Ottenhoff TH, van Dissel JT, Aren SM. Effect of tuberculin testing on a *Mycobacterium tuberculosis*-IFN-γ assay. Eur Respir J 2007 Jan 10;
34. Leger LA, Lambert J. A maximal multistage 20-m shuttle run test to predict VO2 max. European journal of applied physiology and occupational physiology. 1982; 49(1): 1-12.
35. Melo X, Santa-Clara H, Almeida JP, Carnero EA, Sardinha LB, Bruno PM, et al. Comparing several equations that predict peak VO2 using the 20-m multistage-shuttle run-test in 8-10-year-old children. European journal of applied physiology. 2011; 111(5): 839-49.
36. Styblo K. The relationship between the risk of tuberculous infection and the risk of developing infectious tuberculosisBull Int Union Tuberc Lung Dis 1985;60:117-9
37. Van Leth F, Van der Werf MJ, Borgdorff MW (2008) Prevalence of tuberculous infection and incidence of tuberculosis; a re-assessment of the Styblo rule.Bull World Health Organ. 2008;86(1):20-6.
38. Maalouf J, Nabulsi M, Vieth R, Kimball S, El-Rassi R, Mahfoud Z, El-Hajj Fuleihan G.. Short- and long-term safety of weekly high-dose vitamin D3 supplementation in school children. J Clin Endocrinol Metab 2008;93, 2693-2701
39. Oliveri B, Cassinelli H, Mautalen C, Ayala M. Vitamin D prophylaxis in children with a single dose of 150000 IU of vitamin D. Eur J Clin Nutr. 1996; 50(12): 807-10

40. Institute of Medicine. Dietary Reference Intakes for Calcium and Vitamin D. Washington, DC: National Academy Press; 2011.
41. Manaseki-Holland S, Maroof Z, Bruce J, Mughal MZ, Masher MI, Bhutta ZA, et al. Effect on the incidence of pneumonia of vitamin D supplementation by quarterly bolus dose to infants in Kabul: a randomised controlled superiority trial. Lancet. 2012; 379(9824): 1419-27.

3. Summary of Changes to the Study Protocol

A total of 16 modifications were made to the trial protocol, from version 1.0 dated 12th February 2015 to version 17.0 dated 25th April 2019. The majority of these modifications related to addition or removal of names of study personnel. However, the following substantive changes to the scientific content of the trial protocol were also made:

A. Addition of the following secondary aims:

1. To determine whether vitamin D supplementation influences the following secondary efficacy outcomes:

a) in all participants:

• incidence of self-reported acute respiratory infection (upper, lower and both combined)
• incidence of acute respiratory infection requiring hospitalization
• incidence of acute respiratory infections requiring antibiotic treatment
• number of days off school (total number and number due to acute respiratory infection)
• incidence of acute asthma exacerbation requiring hospitalization
• incidence of new asthma, allergic rhinitis and atopic dermatitis arising since baseline, based on ISAAC questionnaire data
• control of asthma, allergic rhinitis and atopic dermatitis identified at baseline, based on ISAAC questionnaire data
• incidence of bone fracture
• anthropometric outcomes (z-scores for height-for-age, weight-for-age, weight-for-height, body mass index-for-age, and waist circumference and waist-to-height ratio)
• body composition: impedance, impedance%, fat mass fat %, and fat-free mass
• muscle strength: grip strength and long jump distance from standing
• serum 25-hydroxyvitamin D concentration

b) in a sub-set of participants:

• bone mineral density (BMD) at the radius
• physical fitness (maximal oxygen consumption estimated from 20m shuttle run)
• attention-related behavior scores (Connors III)
• prevalence of dental caries
• circulating and antigen-stimulated concentrations of cytokines, chemokines and other inflammatory mediators
• exam performance
• self-reported pubertal development
• urinary metabolome profile
• spirometric lung volumes (FEV1 and FVC)
• gut microbiome profile

2. To determine whether any effects of vitamin D supplementation on the outcomes above are modified by calcium intake or genetic variation in the vitamin D pathway (secondary outcome, efficacy).

3. To confirm that the regimen of vitamin D supplementation described above is safe and well-tolerated (secondary outcomes, safety)

4. To determine whether vitamin D supplementation is cost-effective for the prevention of LTBI and active TB.

B. Revision of sample size calculations:

1. Main study

We originally estimated that we would need to randomize a total of 8020 subjects (4010 per arm) to detect a 25% reduction in the proportion of participants acquiring tuberculosis infection with 80% power and 5% alpha, assuming a 2% annual risk of tuberculosis infection, 10% loss to follow-up and an additional 5% loss due to indeterminate QFT-G results at the end of the study. Subsequently, we became

concerned that rates of loss to follow-up might be higher than originally anticipated, and consequently the target sample size for the main trial was increased to 8850.

2. BMD/spirometry sub-study

We originally estimated that we would need to recruit a total of 952 participants (476 per arm) to the BMD sub-study in order to detect a difference in radial BMD of 0.2 z-scores between study arms with 80% power and 5% alpha, assuming standard deviation for 3-year BMD z-score of 1.1 and 20% loss to follow-up. Subsequently, we became concerned that rates of loss to follow-up might be higher than originally anticipated, and consequently the target sample size for this sub-study was increased to 1464.

3. Fitness sub-study

We originally estimated that we would need to recruit a total of 420 participants (210 per arm) to detect a difference in maximal rate of oxygen consumption (VO2max) of 2 ml/kg/min between study arms with 80% power and 5% alpha, assuming standard deviation for 3-year VO2max of 6.5 ml/kg/min and 20% loss to follow-up. Subsequently, we became concerned that rates of loss to follow-up might be higher than originally anticipated, and consequently the target sample size for this sub-study was increased to 614.

C. Addition of the following study assessments and procedures:

- Measurements of waist circumference (all participants at baseline, 12 months, 24 months and 36 months)
- Long jump test (all participants at baseline, 12 months, 24 months and 36 months)
- Measurement of radial BMD (n=1464 sub-set of participants at baseline, 12 months, 24 months and 36 months)
- Fitness test (n=614 sub-set of participants at baseline, 12 months, 24 months and 36 months)

• Attention Deficit Disorder/Attention Deficit Hyperactivity Disorder test (n=201 subset sub-set of participants at baseline and 36 months)
• Assessment of dental caries (n=220 sub-set of participants at baseline and 36 months)
• Tanner assessment of pubertal development (n=1200 sub-set of participants at 36 months)
• Calcium intake questionnaire (all participants, at 24, 27, 30, 33 and 36 months)
• Fracture questionnaire (all participants, at 24, 27, 30, 33 and 36 months)
• Dietary questionnaire (n=1200 sub-set of participants at 36 months)
• Collection of random spot urine sample (n=1200 sub-set of participants at 36 months)
• Spirometry (n=1200 sub-set of participants at 36 months)
• Collection of fecal sample (n=285 sub-set of participants at 36 months)

D. Changes to the Statistical Analysis Plan:

• Pre-specification of analysis of QFT conversion at the 4.0 IU/ml IFN-γ threshold was added
• Pre-specification of analysis of incident active TB as assessed by end-point committee was added (in addition to incident active TB as diagnosed by local clinicians).
• The analytic approach for the outcome of active TB disease was changed from survival analysis of time to event using Cox regression to comparison of proportions developing active TB, using Mantel-Haenzsel risk ratio stratified by school.
• Addition of sub-group analyses, specifying that efficacy analyses may be repeated to include:

a) An interaction term between baseline vitamin D status and allocation to vitamin D vs. placebo

b) An interaction term between vitamin D pathway genotype and allocation to vitamin D vs. placebo (subject to availability of funding)

c) An interaction term between estimated calcium intake and allocation to vitamin D vs. placebo
